# Inhibition of MELK produces potential anti‐tumour effects in bladder cancer by inducing G1/S cell cycle arrest via the ATM/CHK2/p53 pathway

**DOI:** 10.1111/jcmm.14878

**Published:** 2019-12-10

**Authors:** Song Chen, Qiang Zhou, Zicheng Guo, Yejinpeng Wang, Lu Wang, Xuefeng Liu, Mengxin Lu, Lingao Ju, Yu Xiao, Xinghuan Wang

**Affiliations:** ^1^ Department of Urology Zhongnan Hospital of Wuhan University Wuhan China; ^2^ Department of Urology Enshi Clinical College of Wuhan University Enshi China; ^3^ Department of Urology Qingdao Municipal Hospital Qingdao University Qingdao China; ^4^ Department of Pathology Lombardi Comprehensive Cancer Center Georgetown University Medical Center Washington DC USA; ^5^ Department of Biological Repositories Zhongnan Hospital of Wuhan University Wuhan China; ^6^ Laboratory of Precision Medicine Zhongnan Hospital of Wuhan University Wuhan China; ^7^ Human Genetics Resource Preservation Center of Hubei Province Wuhan China; ^8^ Medical Research Institute Wuhan University Wuhan China

**Keywords:** bladder cancer, cell cycle, MELK, OTSSP167, p53

## Abstract

We aimed to investigate the biological function of MELK and the therapeutic potential of OTSSP167 in human bladder cancer (BCa). First, we observed overexpression of MELK in BCa cell lines and tissues and found that it was associated with higher tumour stage and tumour grade, which was consistent with transcriptome analysis. High expression of MELK was significantly correlated with poor prognosis in BCa patients, and MELK was found to have a role in the cell cycle, the G1/S transition in mitosis, and DNA repair and replication. Furthermore, BCa cells presented significantly decreased proliferation capacity following silencing of MELK or treatment with OTSSP167 in vitro and in vivo. Functionally, reduction in MELK or treatment of cells with OTSSP167 could induce cell cycle arrest and could suppress migration. In addition, these treatments could activate phosphorylation of ATM and CHK2, which would be accompanied by down‐regulated MDMX, cyclin D1, CDK2 and E2F1; however, p53 and p21 would be activated. Opposite results were observed when MELK expression was induced. Overall, MELK was found to be a novel oncogene in BCa that induces cell cycle arrest via the ATM/CHK2/p53 pathway. OTSSP167 displays potent anti‐tumour activities, which may provide a new molecule‐based strategy for BCa treatment.

**Abbreviations**
AML: acute myeloid leukaemia
AMPK: AMP protein kinase
ATM: ataxia telangiectasia‐mutated
AUC: areas under the curve
BCa: bladder cancer
CHK2: cell cycle checkpoint kinase 2
CSS: cancer‐specific survival
DAVID: Database for Annotation, Visualization and Integrated Discovery
DIPG: diffuse intrinsic pontine glioma
DSBs: double‐strand breaks
EMT: epithelial to mesenchymal transition
FBS: foetal bovine serum
GEO: gene expression omnibus
GO: gene ontology
GSEA: gene set enrichment analysis
KEGG: Kyoto Encyclopedia of Genes and Genomes
MELK: maternal embryonic leucine zipper kinase
MIBC: muscle‐invasive bladder cancer
MSigDB: Molecular Signatures Database
NMIBC: non‐muscle‐invasive bladder cancer
OS: overall survival
PCR: polymerase chain reaction
qRT‐PCR: quantitative real‐time polymerase chain reaction
ROC: receiver operating characteristic
SD: standard deviation

## 1 INTRODUCTION

Bladder cancer (BCa) is a common urinary tumour that is biologically and clinically heterogeneous.^1^, ^2^ The recurrence of non‐muscle‐invasive bladder cancer (NMIBC) is very frequent; nevertheless, once it progresses into muscle‐invasive bladder cancer (MIBC), the prognosis is poor.^2^, ^3^ Defects in the p53 pathway contribute to diagnosis and therapy difficulties as well as adverse clinical outcome in BCa, which represents an urgent clinical need with few breakthroughs in treatment in the last ten years.^4^, ^5^

Maternal embryonic leucine zipper kinase (MELK) is a member of the AMP protein kinase (AMPK) family of serine/threonine kinases, and MELK activates multiple cellular pathways that drive oncogenic growth.^6^, ^7^, ^8^ MELK was associated with mitotic progression and DNA damage.^8^, ^9^, ^10^, ^11^, ^12^, ^13^ It has been shown that MELK is overexpressed in multiple human tumours, including the following: melanoma,^8^ diffuse intrinsic pontine glioma (DIPG),^14^ breast cancer,^6^, ^15^ gastric cancer,^16^ high‐grade prostate cancer,^17^ hepatocellular carcinoma,^18^ kidney cancer,^19^ small lung cancer,^20^ myeloma,^21^ acute myeloid leukaemia (AML)^22^ and chronic lymphocytic leukaemia (CLL).^23^ High levels of MELK are correlated with clinically aggressive disease and poor survival.^15^, ^23^, ^24^ Further, genomic or pharmacologic inhibition of MELK has been shown to suppress tumour growth in vitro and in pre‐clinical adult cancer models, indicating that this kinase is a potential therapeutic target.^19^, ^22^, ^23^, ^24^, ^25^ A number of studies have shown that MELK inhibition also increases sensitivity to radiation and chemotherapy in pre‐clinical adult cancer models, suggesting that combination treatments may also be effective strategies.^23^, ^26^, ^27^, ^28^, ^29^ Although the mechanisms by which MELK mediates aggressive tumour growth are not completely understood, MELK inhibition has been confirmed to lead to the consecutive phosphorylation of ataxia telangiectasia‐mutated (ATM) and cell cycle checkpoint kinase 2 (CHK2) and to the up‐regulation of p53 and p21.^13^, ^23^, ^25^, ^30^, ^31^, ^32^

OTSSP167, a type I kinase inhibitor, is an inhibitor that markedly suppresses MELK kinase activity by phosphorylating the MELK substrates DBNL and PSMA1.^23^ The potent anti‐tumour effects of OTSSP167 have been observed in multiple malignant tumours, such as breast cancer,^24^ myeloma^21^ and AML.^23^ Several phase I/II clinical trials in patients with advanced breast cancer and acute myeloid leukaemia have been launched to test the therapeutic potential of OTSSP167.^23^, ^25^ Nevertheless, the biological function of MELK and the therapeutic potential of OTSSP167 in BCa are still unclear. Interestingly, MELK was implicated in tumorigenesis via the p53 pathway,^13^, ^23^, ^25^, ^30^, ^31^, ^32^ which suggested the potential utility of OTSSP167 in treating relapsed/refractory BCa. Therefore, we aimed to investigate the biological function of MELK and the therapeutic potential of OTSSP167 in BCa.

Herein, we conducted a study to investigate the expression and prognostic significance of MELK in BCa for the first time. Loss‐of‐function and gain‐of‐function assays were performed to investigate the biological roles of MELK and unravel the regulatory mechanism of MELK and OTSSP167 in BCa. Bioinformatics analyses confirm our study results and our hypothesis. Altogether, MELK is a novel oncogene involved in BCa‐induced cell cycle arrest via the ATM/CHK2/p53 pathway. OTSSP167 displays potent anti‐tumour activities in BCa cells and nude mouse transplantation tumour experiments, which may provide a new molecule‐based strategy for BCa treatment.

## 2 MATERIALS AND METHODS

### 2.1 Clinical specimens and cell lines

The tumour and paracancerous tissue samples (n = 31) in this study were obtained from radical cystectomy patients at Zhongnan Hospital of Wuhan University. In addition, regarding the data from another cohort study (n = 2), the tissues were collected from transurethral resection bladder tumour (newly diagnosed tumour) and radical cystectomy (progression tumour). The clinical, pathological and follow‐up data records of all patients were collected, and two pathologists were invited to independently confirm the histology diagnosis. The study using clinical information and surgical tissue specimens was approved by the Ethics Committee of Zhongnan Hospital of Wuhan University (approval number: 2 015 029). All patients provided informed consent. The procedures in this study were performed in accordance with the ethical standards of the institutional and/or national research committee.

We purchased the human epithelial SV40 immortalized uroepithelium cell line SV‐HUC‐1 as well as human BCa cell lines (T24, J82, UMUC3, RT4 and 5637) from the Stem Cell Bank, Chinese Academy of Sciences in Shanghai, China. The China Centre for Type Culture Collection recognizes these as BCa cell lines. SV‐HUC‐1, T24 and 5637 cell lines were cultured in RPMI‐1640 medium (Gibco), J82 cells were cultured in MEM (Gibco), RT4 cells were maintained in McCoy's 5 A Medium (Gibco), and UMUC3 cells were cultured in DMEM with high glucose (Gibco) and 10% foetal bovine serum (FBS) (Gibco). These cell lines were grown at 5% CO_2_ and 37°C in a humidified incubator (Thermo Scientific). All human cell lines have been authenticated using STR profiling within the last three years. All cell lines used are listed using the official cell line name and its research resource identifier (RRID) as available in the ExPASy Cellosaurus database. In addition, the source/suppliers of all cell lines used have been provided. All experiments were performed with mycoplasma‐free cells.

### 2.2 Bioinformatics analyses

In addition to the data from our centre, we also accessed the microarray expression profiles of http://www.ncbi.nlm.nih.gov/geo/query/acc.cgi?acc=GSE13507 from gene expression omnibus (GEO) to obtain clinical and cytogenetic data of external BCa patients. Database for Annotation, Visualization and Integrated Discovery (DAVID) Bioinformatics Resources 6.8 (https://david.ncifcrf.gov) helped reveal the biological processes and signal pathways that MELK is involved in BCa tumorigenesis. The cut‐off was set as *P* < .05. Gene set enrichment analysis (GSEA) software was used to analyse the correlation between MELK expression and hallmark gene sets from the Molecular Signatures Database (MSigDB).

### 2.3 Transfections and stable cell line selection

*MELK‐siRNA*s (*si‐MELK*s) and *control‐siRNA* (NC) oligonucleotides were synthesized by GenePharma Gene Co Ltd. *MELK‐shRNA* (*sh‐MELK*) was synthesized by JTSBIO, Ltd. The sense sequence of *MELK‐siRNA‐1* (*si‐1*)/*sh‐MELK* was 5’‐CCUGGAUCAUGCAAGAUUATT‐3’, the sense sequence of *MELK‐siRNA‐2* (*si‐2*) was 5’‐GGCGGGAUUAAUAGACUAUT‐T3’, the sense sequence of *MELK‐siRNA‐3* (*si‐3*) was 5’‐GCCAAAGACUCCAGUU‐AAUTT‐3’, and the sense sequence of *control‐siRNA* (NC)*control‐shRNA* (NC) was 5’‐UUCUCCGAACGUGUCACGUTT‐3’. MELK cDNA (1832 bp) was polymerase chain reaction (PCR) amplified from a cDNA library of human BCa cell lines and then cloned into a 2 × FIagpcDNA3 empty vector performed with a one‐step method to construct the homologous recombination vectors. The MELK forward primer sense sequence was 5’‐GATAAAGGTCACCCAATGAAAGATTATGATGAACTTC3’, and the MELK reverse primer sense sequence was 5’‐TGATGGATATCTGCATTATACCT‐TGCAGCTAGATAGG‐3’. According to the manufacturer's protocol, cells were transfected with plasmids or siRNA oligonucleotides using Lipofectamine 2000 (Invitrogen) transfection reagent. To select stable cell lines, UMUC3 cells were infected with *MELK‐shRNA* and *control‐shRNA*. After 24 hours, 5 μg/mL puromycin (Sigma‐Aldrich) was added to the cell culture medium, and it was used to select cells for 14 days. The antibiotic‐resistant cells were successfully selected.

### 2.4 RNA isolation, reverse transcription and quantitative real‐time PCR (qRT‐PCR)

Following the manufacturer's protocol, a HiPure Total RNA Mini Kit (Cat. #R4111‐03, Magen) was used to isolate total RNA from cells and bladder tissues. We performed reverse transcription with a ReverTra Ace qPCR RT Kit (Toyobo). qRT‐PCR was conducted with iQTM SYBR^®^ Green Supermix (Bio‐Rad). Table S1 lists the primer sequences used. Fold enrichment was calculated with the 2^−ΔΔCt^ method relative to the expression of GAPDH.

### 2.5 Flow cytometry analysis

The transfected BCa cells were gathered, centrifuged and washed twice with cold PBS. Then, we resuspended the cell precipitation with a 1 × DNA staining solution containing propidium iodide and permeabilization solution (Multi Sciences) in the dark and incubated the mix at 37°C for 30 minutes. Flow cytometry (Cat. #FC500, Beckman) was used to analyse the cell cycle distribution.

### 2.6 Pre‐treatment of OTSSP167

T24 and UMUC3 cells were first grown under normal conditions for 24 hours and were subsequently treated with OTSSP167 (dissolved in 1% DMSO, Cat. #S7159, Selleck Chemicals) at final concentrations of 5, 10, 15, 30, 35, 40, 45 and 50 nM for 24, 48 and 72 hours to identify the appropriate concentrations. All the following relevant experiments were conducted with the cells pre‐treated with OTSSP167 at 0, 10 and 30 nM for 48 hours. Control BCa cells were also pre‐incubated with 0.1% DMSO for the same amounts of time. In contrast, for the restoration experiments, T24 and UMUC3 cells were transfected with a plasmid for 72 hours after pre‐treatment with OTSSP167 for 12 hours.

### 2.7 Total protein extraction and Western blot analysis

Using PBS, BCa cells were washed 3 times and then were lysed for 30 minutes on ice with a solution that contained RIPA buffer, protease inhibitor and phosphatase inhibitor (Sigma‐Aldrich). The cell lysates were centrifuged at 13 000 × g for 15 minutes, and a Bradford protein assay (Bio‐Rad) was used to determine the protein concentration of the collected supernatants. Western blot analysis was performed after the total protein samples were separated by 7.5%‐15% SDS‐PAGE. Immunoreactive bands were visualized with an enhanced chemiluminescence kit (Bio‐Rad) and were then detected with a Molecular Imager ChemiDoc XRS + Imaging system (Bio‐Rad). Table S2 and Table S3 list the primary antibodies and secondary antibodies used, respectively.

### 2.8 Xenograft mouse model

We purchased BALB/c‐nu mice (male, 3 weeks old) from Beijing Vital River Laboratory Animal Technology Co., Ltd. The laboratory animal facility of Zhongnan Hospital of Wuhan University housed and fed the animals. The animal experiments were performed in accordance with institutional guidelines. UMUC3 NC cells (1 × 10^6^) or *sh‐MELK* cells diluted in 100 μL PBS (n = 6) were subcutaneously injected to establish xenograft models after mice were adaptively fed for 1 week. For the OTSSP167 injection anti‐tumour experiment, mice were subcutaneously inoculated with 1 × 10^6^ UMUC3 cells diluted in 100 μL PBS (n = 12). Subsequently, tumour volume was measured every 3 days (tumour volume = length ×width × 0.5 mm^3^). We killed the mice 6 weeks later, after which we removed the tumours and then weighed them.

### 2.9 Statistical analyses

The data were expressed as the mean ± standard deviation (SD) of three individual experiments. All continuous measures were compared by a two‐sample t tests. A receiver operating characteristic (ROC) curve was generated for the MELK mRNA level to calculate the areas under the curve (AUC). The highest Youden's index, which was established as the optimized point, was used to determine the optimal cut‐off for MELK mRNA levels based on the ROC curve. The associations between the MELK expression level and the clinicopathological factors in BCa patients were analysed with chi‐squared tests. Kaplan‐Meier curves were generated to estimate overall survival (OS) and cancer‐specific survival (CSS), and log‐rank tests were used to assess survival differences among subgroups. The expression of MELK, age, gender, T stage, N stage, M stage, tumour grade, recurrence and progression were used as covariates, and Cox univariate and multivariate survival analyses were performed to estimate independent prognostic factors associated with patient survival. Nomograms were generated based on Cox regression analyses. Calibration curves were generated to assess the agreements of the nomogram‐predicted probability with the actual observed probability. We used SPSS 16.0 and GraphPad Prism 7 to perform all statistical analyses. Nomograms and calibration curves were generated with R version 3.5.0, and a *P* value < .05 was considered statistically significant.

## 3 RESULTS

### 3.1 MELK was overexpressed in BCa patients and associated with poor prognosis as well as progression

**Figure 1:**
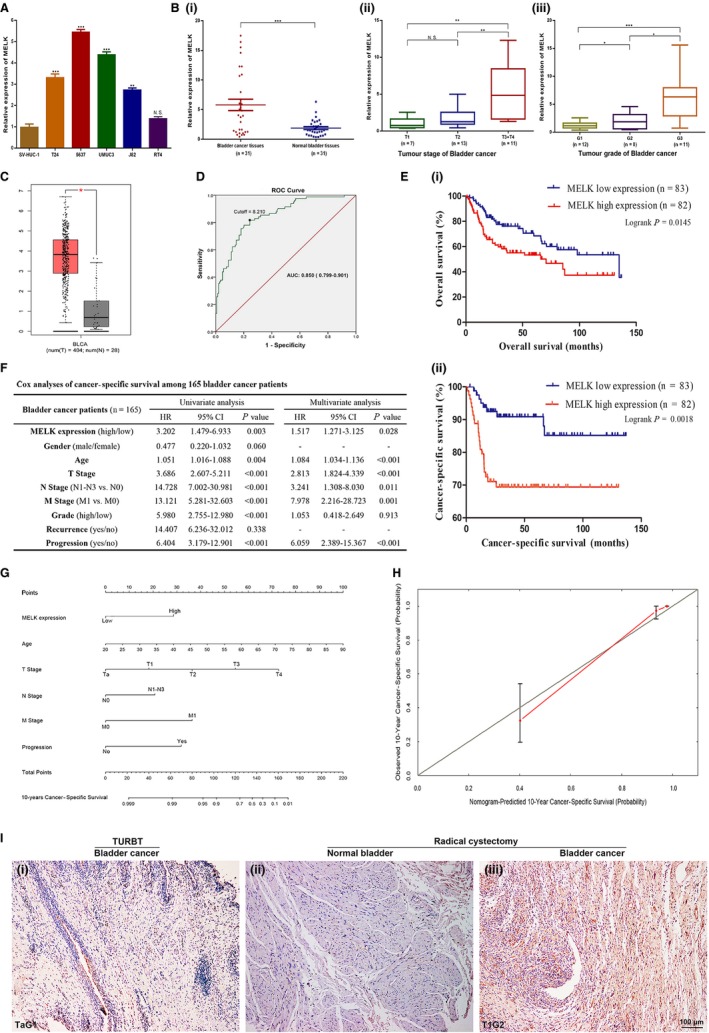
MELK was overexpressed in BCa patients and was associated with poor prognosis and progression. A, MELK mRNA was significantly overexpressed in BCa cell lines (T24, 5637, UMUC3, J82) compared with the normal bladder epithelial cell line (SV‐HUC‐1). B, MELK mRNA was overexpressed and positively related to the tumour stage and tumour grade in the BCa tissues. C, In the TCGA database, MELK mRNA was overexpressed in BCa. D, The cut‐off value of MELK relative expression level was determined to be 8.210 by the ROC curve. E, Kaplan‐Meier survival (OS and CSS) curves of BCa patients from http://www.ncbi.nlm.nih.gov/geo/query/acc.cgi?acc=GSE13507 with stratified MELK expression. F, Cox analyses of cancer‐specific survival among 165 bladder cancer patients. G, The nomogram for 10‐year cancer‐specific survival prediction of BCa patients. H, The calibration curves were developed for the 10‐year cancer‐specific survival prediction nomogram. I, IHC staining is shown for MELK in radical cystectomy BCa tissues and paired paracancerous tissues as well as the transurethral resection bladder tumour tissues in a cohort study, * *P* < .05; ** *P* < .01; *** *P* < .001

MELK mRNA was analysed by qRT‐PCR to investigate the expression level in BCa. Compared with SV‐HUC‐1 cells, the MELK mRNA expression level was significantly higher in BCa cell lines (all *P* < .01 except RT4, Figure 1A). The qRT‐PCR analysis also indicated that MELK mRNA was more highly expressed in BCa tissues than in normal tissues (n = 31). Table S4 summarizes the clinicopathological information of the 31 BCa patients. Combined with the clinicopathological information, further analysis indicated that the expression levels of MELK mRNA were positively related to the tumour stage and tumour grade in BCa (Figure 1B).

We re‐evaluated the TCGA database and a genomic microarray profile http://www.ncbi.nlm.nih.gov/geo/query/acc.cgi?acc=GSE13507 (n = 165) to reduce the limitation of sample size and racial differences. The TCGA database (404 BCa tissues and 28 normal bladder tissues) confirmed at the mRNA level the MELK expression results we observed in our tissue samples (Figure 1C). In http://www.ncbi.nlm.nih.gov/geo/query/acc.cgi?acc=GSE13507, with the method mentioned, the cut‐off value of MELK relative expression level was determined to be 8.210 (AUC = 0.850, Figure 1D). High expression was defined when the expression level was greater than 8.210. In the Kaplan‐Meier survival analyses, compared with BCa patients with low MELK, patients with high MELK had decreased OS and CSS (log‐rank, *P* = .0145, *P* = .0018, respectively, Figure 1E). Chi‐square testing showed that the MELK level was significantly correlated with gender, age, T stage, N stage, tumour grade and progression (Table 1). In addition, univariate and multivariate Cox analyses (Figure 1F and Figure S1A) showed that high expression of MELK predicted poor prognosis (CSS and OS, HR = 1.517, HR = 1.384, respectively, both *P* < .05). Based on the Cox regression analyses, nomograms were constructed to calculate the survival probability for each BCa patient directly. The 10‐year CSS (Figure 1G) and OS (Figure S1B) probabilities of BCa patients could be accurately calculated via the nomogram. The calibration curves displayed agreements of the nomogram‐predicted probability with the actual probability for CSS (Figure 1H) and OS (Figure S1C).

**Table 1:** Association between MELK expression and clinicopathological features of human bladder cancer

Variables	MELK expression in human bladder cancer tissues				
	Cases (n = 165)	Low(n = 83)	High(n = 82)	χ^2^	*P* value
Gender, n (%)				4.224	.040
Male	135 (81.8)	73 (88.0)	62 (75.6)		
Female	30 (18.2)	10 (12.0)	20 (24.4)		
Age (years), n (%)				6.849	.009
<65	69 (41.8)	43 (51.8)	26 (31.7)		
≥65	96 (58.2)	40 (48.2)	56 (68.3)		
T stage, n (%)				14.205	<.001
Ta‐T1	104 (63.0)	64 (77.1)	40 (48.8)		
T2‐T4	61 (37.0)	19 (22.9)	42 (51.2)		
N stage, n (%)				7.056	.008
N0	149 (90.3)	80 (96.4)	69 (84.1)		
N1‐N3	16 (9.7)	3 (3.6)	13 (15.9)		
M stage, n (%)				‐	.720
M0	158 (95.8)	80 (96.4)	78 (95.1)		
M1	7 (4.2)	3 (3.6)	4 (4.9)		
Tumour grade, n (%)				30.928	<.001
Low	105 (63.6)	70 (84.3)	35 (42.7)		
High	60 (36.4)	13 (15.7)	47 (57.3)		
Recurrence, n (%)				0.025	.875
No	67 (65.0)	42 (65.6)	25 (64.1)		
Yes	36 (35.0)	22 (34.4)	14 (35.9)		
Progression, n (%)				4.972	.026
No	134 (81.2)	73 (88.0)	61 (74.4)		
Yes	31 (18.8)	10 (12.0)	21 (25.6)		

To further confirm the correlations between MELK expression and bladder tumour progression, we tracked two newly diagnosed BCa patients whose tumours occurred within one year. As a cohort study, the tissues were collected from transurethral resection bladder tumour (newly diagnosed tumour) and radical cystectomy (progression tumour) and then performed immunohistochemistry staining to detect MELK expression in progressing bladder tumours. Patient A was a 62‐year‐old man with a TaG1 BCa (unifocal tumour) when first diagnosed, and the tumour progressed into a T1G2 BCa (multifocal tumours) 10.3 months later. Patient B was a 53‐year‐old man with a T1G1 BCa (unifocal tumour) when first diagnosed, and the tumour progressed into a T2G2 BCa (unifocal tumour) 8.9 months later. The IHC staining results showed that MELK expression in the radical cystectomy BCa tissues was significantly up‐regulated compared with that in the paired paracancerous tissues and the transurethral resection bladder tumour tissues. Further, MELK expression increased in concert with tumour progression (Figure 1I).

### 3.2 Bioinformatics analyses of MELK in http://www.ncbi.nlm.nih.gov/geo/query/acc.cgi?acc=GSE13507

**Figure 2:**
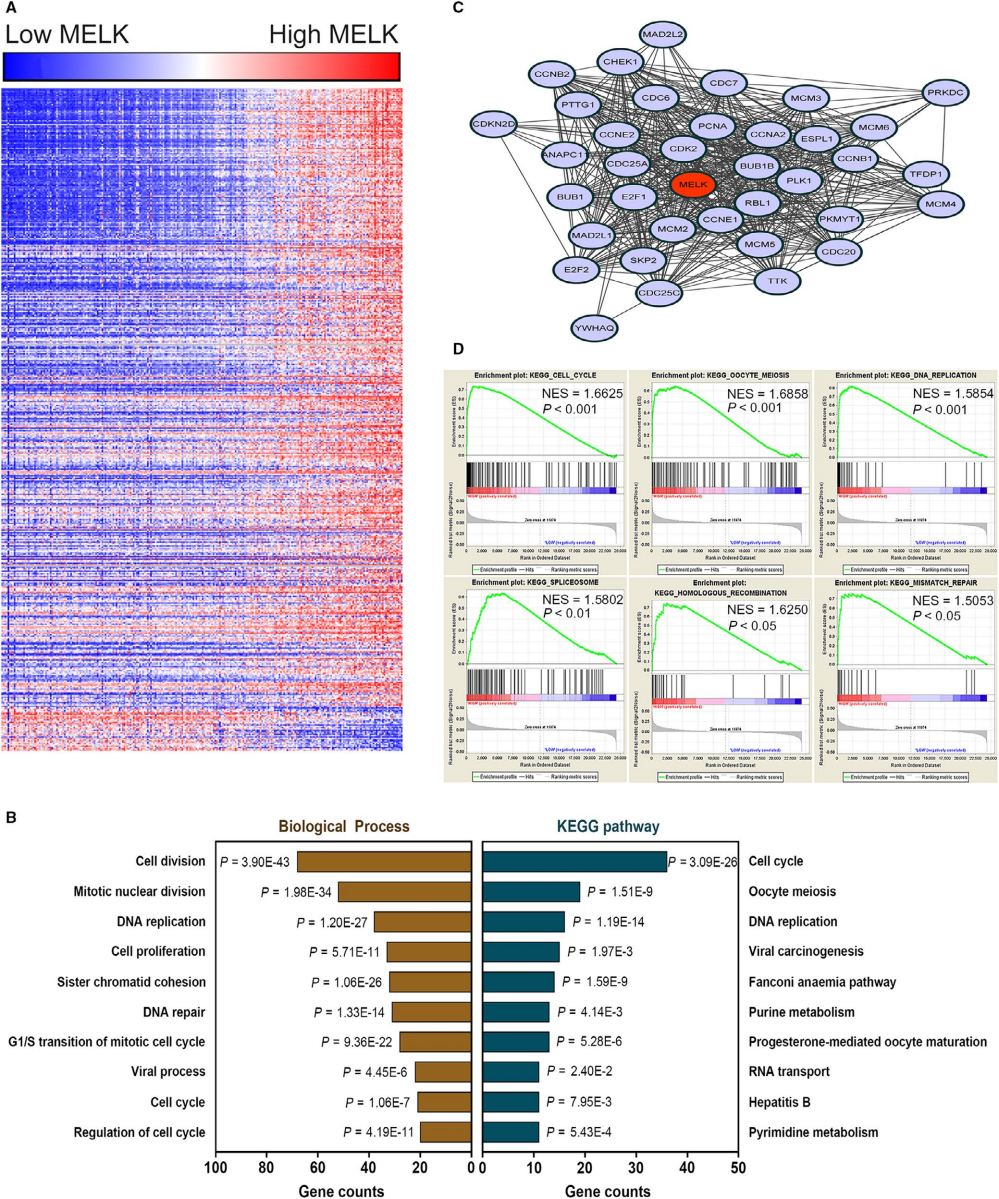
Bioinformatics analysis of MELK in BCa microarray expression profiles. A, Heat map of the MELK‐related gene expression signature in http://www.ncbi.nlm.nih.gov/geo/query/acc.cgi?acc=GSE13507. B, Functional enrichment analyses of MELK expression in http://www.ncbi.nlm.nih.gov/geo/query/acc.cgi?acc=GSE13507. C, Gene‐gene interaction network analysis shows MELK and 36 genes (CDK2, E2F1, CCNE1, etc) that had the most connectivity in BCa. D, GSEA highlights the positive correlation of increased MELK expression with the cell cycle, oocyte meiosis, DNA replication, the spliceosome, recombination and mismatch repair. *NES* normalized enrichment score

To investigate the functional enrichment of MELK in BCa, a relation analysis was performed of MELK expression and the top 500 correlated genes in genomic profiles from http://www.ncbi.nlm.nih.gov/geo/query/acc.cgi?acc=GSE13507. We identified 467 positively related genes and 33 negatively related genes by functional enrichment analyses (Figure 2A and Related file 1). MELK was revealed to be inseparably correlated with biological processes such as cell division, mitotic nuclear division, DNA replication, DNA repair and G1/S transition of the mitotic cell cycle by gene ontology (GO) analysis (Figure 2B and Related file 2). Kyoto Encyclopedia of Genes and Genomes (KEGG) analysis indicated that MELK was enriched in the cell cycle, oocyte meiosis, DNA replication, viral carcinogenesis and the Fanconi anaemia pathway (Figure 2B and Related file 3). Gene‐gene interaction network analysis revealed MELK and 36 genes (CDK2, E2F1, CCNE1, etc) that had the most connectivity in BCa (Figure 2C). GSEA revealed that MELK was functionally enriched in the cell cycle, oocyte meiosis, DNA replication, the spliceosome, recombination and mismatch repair (Figure 2D, Table S5).

Thus, it was discovered that MELK potentially contributes to BCa tumorigenesis by regulating several oncogenic signalling pathways and biological processes, especially the cell cycle.

### 3.3 Reduced expression of MELK repressed BCa cell proliferation and migration

**Figure 3:**
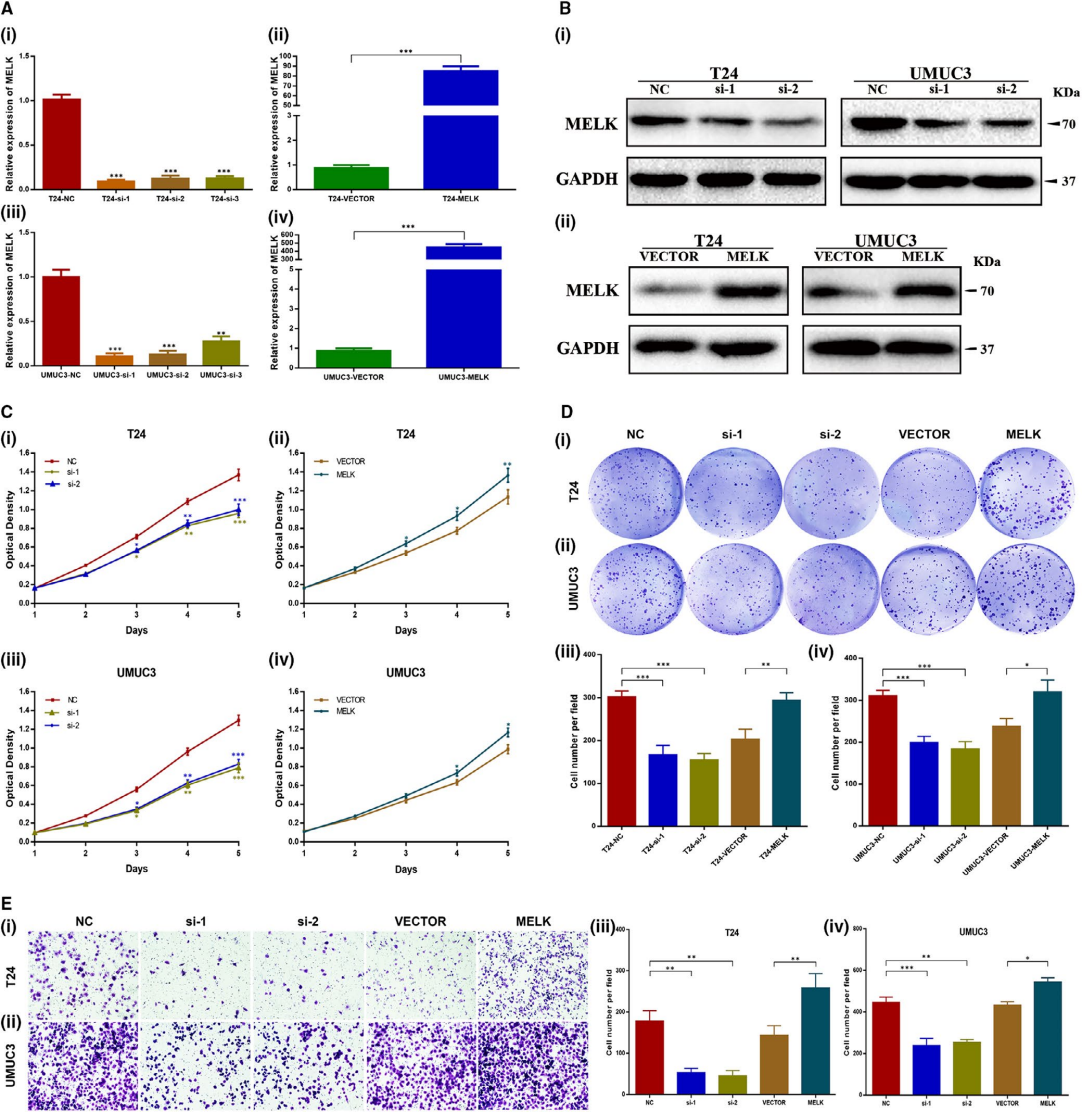
Reduced expression of MELK repressed BCa cell proliferation and migration. A, Verification of *MELK‐siRNA* silencing efficacy and MELK plasmid overexpression efficacy at the mRNA level in T24 cells and UMUC3 cells. B, Verification of *MELK‐siRNA* silencing efficacy and MELK plasmid overexpression efficacy at the protein level in T24 cells and UMUC3 cells. C, D, MTT assays and clonogenic forming assays showed that *MELK* silencing decreased the proliferation capacity, whereas MELK overexpression enhanced the proliferation capacity. E, Migration assays showed that *MELK* silencing attenuated cell migration ability, whereas MELK overexpression enhanced cell migration ability, * *P* < .05; ** *P* < .01; *** *P* < .001

We performed knockdown and overexpression functional assays to investigate the biological function of MELK in BCa cells. Three *siRNA*s demonstrated effective silencing of MELK in T24 and UMUC3 cells at the mRNA and protein levels, of which *MELK‐siRNA‐1* (*si‐1*) and *MELK‐siRNA‐2* (*si‐2*) exhibited a higher degree of MELK knockdown. Moreover, the 2xFIagpcDNA3‐MELK plasmid was constructed and demonstrated good efficacy of MELK overexpression at the transcription and translation levels as well (Figure 3A‐B).

MTT assays showed that, compared with the control group, *si‐1‐* and *si‐2*‐transfected BCa cells presented a significantly decreased proliferation capacity (*P* < .001, Figure 3C). In contrast, BCa cells transfected with the MELK plasmid showed enhanced proliferation capacity (*P* < .05, Figure 3C). The clonogenic assay showed that clone size and number were smaller in *MELK‐siRNA*‐treated BCa cells than they were in NC‐treated BCa cells (*P* < .001, Figure 3D); this phenotype was reversed when MELK was overexpressed (*P* < .05, Figure 3D). The migration assay showed that the migration ability of BCa cells was markedly attenuated when MELK was silenced (*P* < .01, Figure 3E). Moreover, if MELK was overexpressed by transfection with the MELK plasmid, the cell migration ability was markedly enhanced (*P* < .05, Figure 3E).

### 3.4 MELK silencing induced cell cycle arrest at the G1/S phase via the ATM/CHK2/p53 pathway in BCa cells

**Figure 4:**
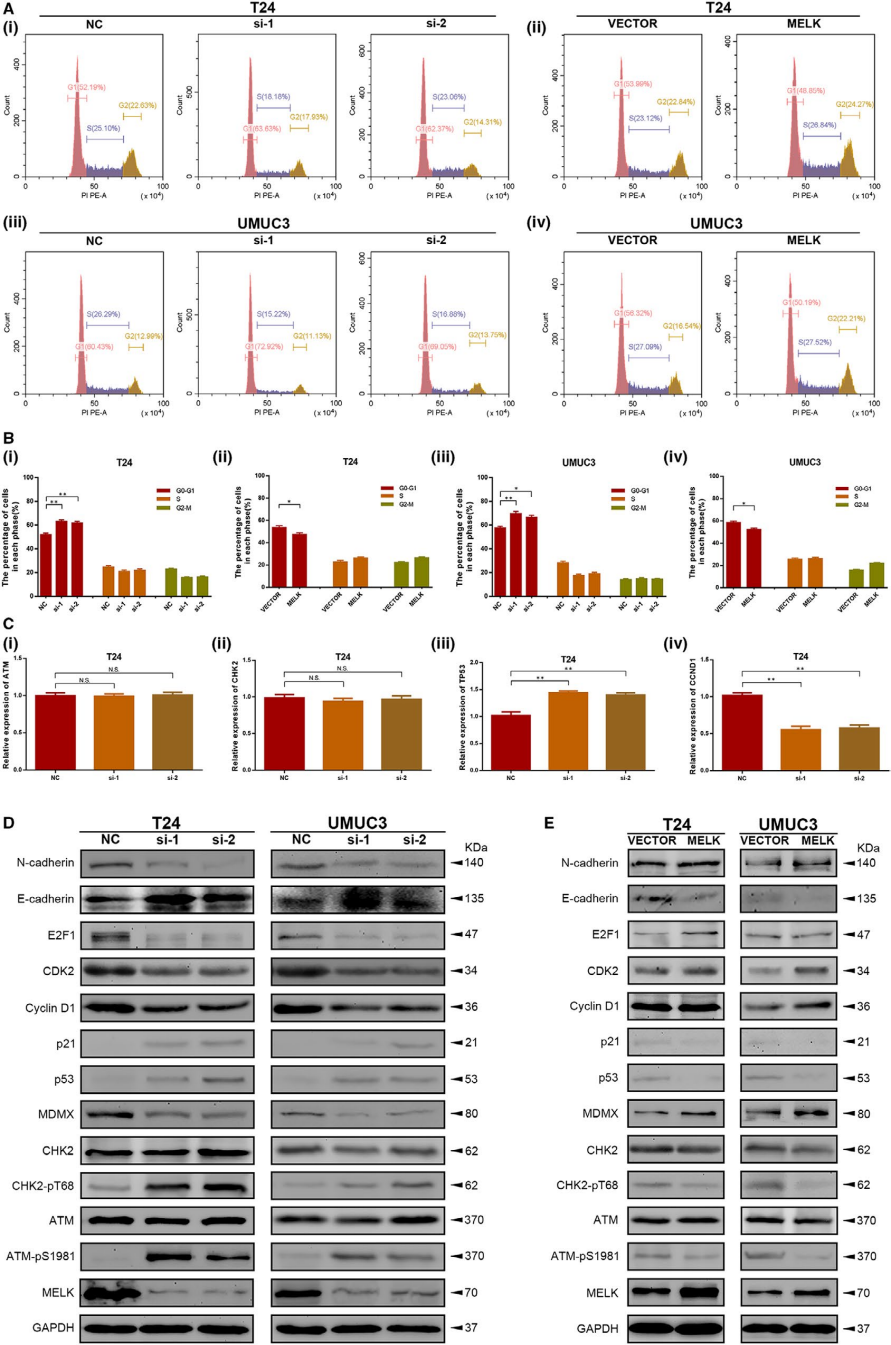
*MELK* silencing induced cell cycle arrest at the G1/S phase via the ATM/CHK2/p53 pathway in BCa cells. A, B, MELK silencing in BCa cells induced cell cycle arrest at the G1/S phase, whereas MELK overexpression in BCa cells caused a decrease in the proportion of cells in the G1/S phase. C, Correlation between MELK, G1/S phase cell cycle‐related genes and ATM/CHK2/p53 pathway‐related genes at the mRNA level in transfected BCa cells was investigated with qRT‐PCR analysis. D, Cell cycle‐related proteins, ATM/CHK2/p53 pathway‐related proteins and EMT proteins were detected in MELK‐silenced BCa cells by Western blot analysis. E, Cell cycle‐related and ATM/CHK2/p53 pathway‐related proteins were detected in MELK‐overexpressing BCa cells by Western blot analysis, * *P* < .05; ** *P* < .01

To validate the above bioinformatics results, flow cytometry analysis was implemented to determine the role of MELK in cell cycle regulation. MELK silencing in BCa cells induced cell cycle arrest at the G1/S phase, which was not observed in the control group. MELK overexpression in BCa cells caused a proportional decrease in the number of cells in the G1/S phase (Figure 4A‐B).

qRT‐PCR analysis was then conducted to investigate the correlation between MELK and G1/S phase cell cycle‐related genes and ATM/CHK2/p53 pathway‐related genes (ATM, CHK2, TP53, CDKN1A, CCND1 and CDK2) in transfected BCa cells. The results demonstrated that MELK could regulate TP53, CDKN1A, CCND1 and CDK2, but MELK did not significantly influence ATM and CHK2 at the transcriptional level (Figure 4C, Figure S2A). Furthermore, Western blot analysis was performed to detect the changes in these cell cycle‐related genes and ATM/CHK2/p53 pathway‐related genes at the protein level. Consistent with previous research, Western blot analysis revealed that down‐regulation of MELK had no significant influence on total ATM and CHK2, but it could promote phosphorylation of ATM and CHK2, suggesting that ATM and CHK2 were inhibited by MELK in BCa cells. In addition, down‐regulation of MELK up‐regulated the expression of p53 and p21, while it reduced MDMX, cyclin D1, CDK2 and E2F1 expression (Figure 4D). When MELK was up‐regulated, phosphorylation of ATM and CHK2 decreased. MELK overexpression increased the expression of MDMX, cyclin D1 and CDK2, E2F1, while it reduced p53 and p21 expression. Moreover, the E‐cadherin and N‐cadherin expression levels detected by Western blot analysis confirmed that MELK promoted BCa cell migration (Figure 4E). Further analysis using the GEPIA database (http://gepia.cancer-pku.cn/index.html) revealed that there was a linear relationship between MELK and these genes, which confirmed our Western blot results (Figure S2B).

Overall, MELK promoted BCa cell vitality by accelerating the transition out of G1/S phase via inhibition of the ATM/CHK2/p53 pathway in BCa cells.

### 3.5 Reduction in MELK suppressed BCa cell growth in vivo

**Figure 5:**
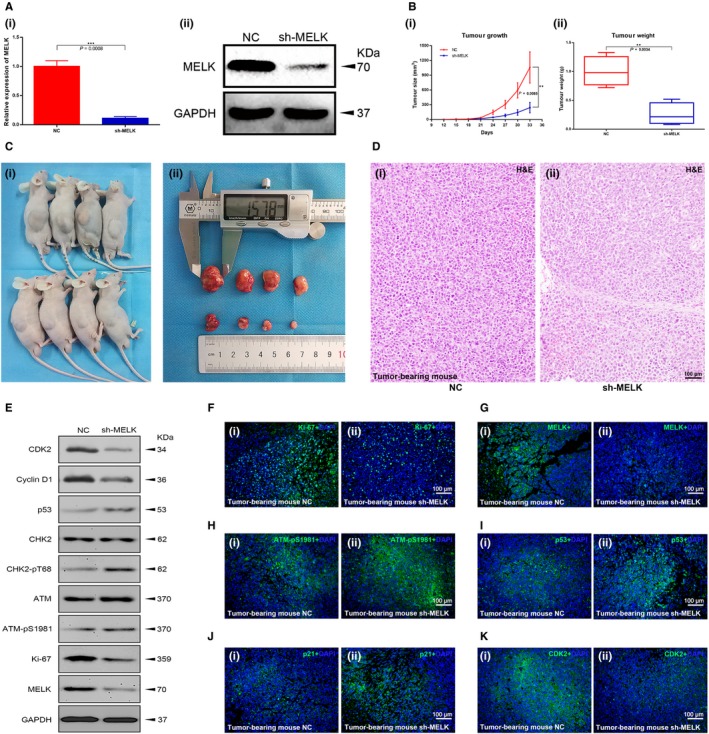
Reduction in MELK suppressed BCa cell growth in vivo. A, The MELK inhibition efficiency of shRNA treatment was confirmed at the transcription and translation levels. B, C, The measurement of tumour growth activity and the weight of dissected tumours. D, H&E, staining of tumour tissues. E, Cell cycle‐related proteins, ATM/CHK2/p53 pathway‐related proteins and Ki‐67 were detected in mouse tumour tissues by Western blot analysis. (F‐K) Cell cycle‐related proteins, ATM/CHK2/p53 pathway‐related proteins and Ki‐67 were detected in mouse tumour tissues by immunofluorescence analysis

A xenograft mouse model was established to investigate the effect of MELK down‐regulation on BCa cell growth in vivo. The MELK inhibition efficiency in stable UMUC3 cells was confirmed by qRT‐PCR and Western blot analysis (Figure 5A). The continuous measurement of tumour growth activity and the weighing of dissected tumours revealed that MELK inhibition significantly suppressed BCa growth in vivo compared with what was observed in control group mice (Figure 5B‐C). H&E staining indicated that the *sh‐MELK* group of tumour tissues contained fewer tumour cells than the tumours in the NC group mice (Figure 5D), suggesting weaker tumour activity in the *sh‐MELK* group.

Total protein and phosphorylated protein levels were measured from the tumour tissues of mice by Western blot analysis. The results further confirmed that MELK inhibited the ATM/CHK2/p53 pathway in vitro (Figure 5E). In addition, immunofluorescence analysis of mouse tumour tissues demonstrated that the number of MELK‐, Ki‐67‐ and CDK2‐positive cells was lower in the MELK inhibition group than it was in the control group, while the number of p‐ATM‐, p53‐ and p21‐positive cells was higher (Figure 5F‐K).

### 3.6 Targeted inhibition of MELK by OTSSP167 caused anti‐tumour activity in BCa cell lines

**Figure 6:**
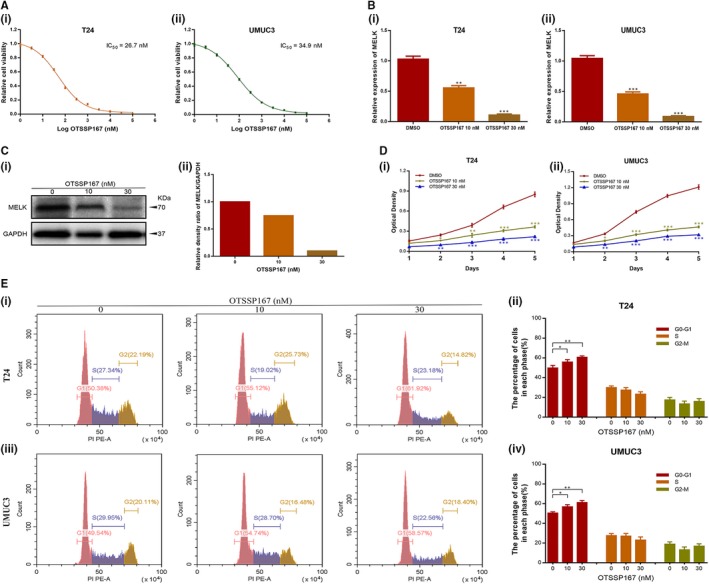
Targeted inhibition of MELK by OTSSP167 resulted in anti‐tumour activity in BCa cell lines. A, The appropriate concentration for OTSSP167 in T24 and UMUC3 cells was selected (IC50 values of 26.74 ± 0.13 nM and 34.88 ± 0.21 nM, respectively). B, C, OTSSP167 decreased MELK expression at the transcription and translation levels in a dose‐dependent manner (0, 10, 30 nM). D, OTSSP167 inhibited the proliferation capacity of T24 and UMUC3 cells at nanomolar concentrations. E, T24 and UMUC3 cells treated with OTSSP167 for 48 h exhibited increases in cells at the G1/S phase, * *P* < .05; ** *P* < .01; *** *P* < .001

OTSSP167 restrained BCa cell line proliferation in a dose‐dependent and time‐dependent manner, with IC50 values of 26.74 ± 0.13 nM in T24 cells and 34.88 ± 0.21 nM in UMUC3 cells (Figure 6A). Thus, 10 nM and 30 nM drug concentrations were selected for the experiments in BCa cell lines. Treatment with OTSSP167 at these two concentration resulted in decreased MELK expression at the mRNA (*P* < .01; Figure 6B) and protein (Figure 6C) levels in a dose‐dependent manner. OTSSP167 also inhibited the proliferation capacity of T24 and UMUC3 cells at nanomolar concentrations (*P* < .05, Figure 6D). In addition, flow cytometric analysis of BCa cell lines treated with OTSSP167 for 48 hours exhibited an increase in cells at the G1/S phase compared with what was observed following DMSO treatment (*P* < .05, Figure 6E). Thus, OTSSP167 exerted therapeutic potential via thwarting BCa cell proliferation and inducing cell cycle arrest in G1/S phase.

### 3.7 Inhibition of MELK with OTSSP167 causes potent anti‐tumour effects in bladder cancer in vivo

**Figure 7:**
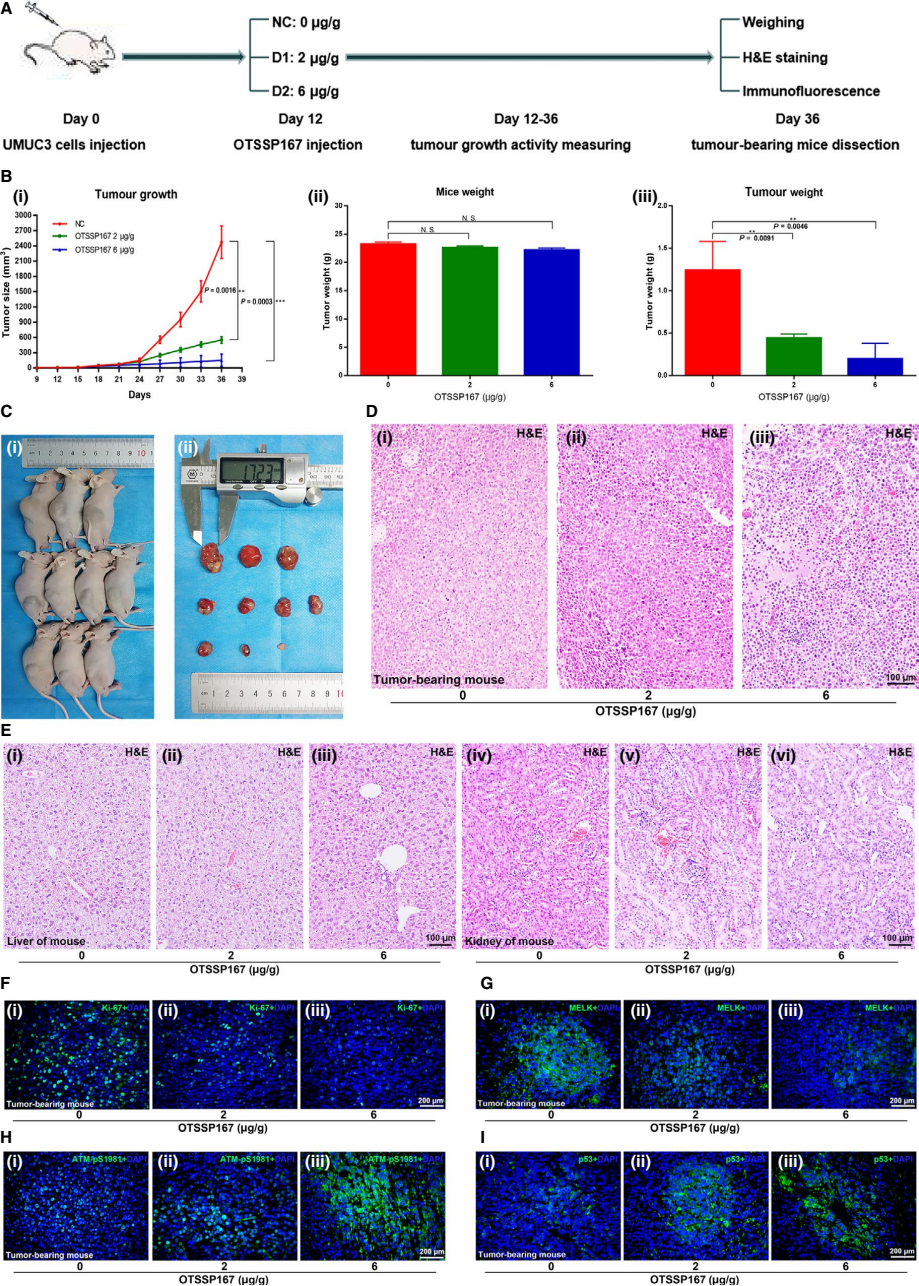
Inhibition of MELK by OTSSP167 results in potent anti‐tumour effects in bladder cancer in vivo. A, The OTSSP167 injection anti‐tumour experiment performed with a mouse xenograft model is shown. B, C, The measurement of tumour growth activity and of mouse bodyweight as well as analysis of the dissected tumours. D, E, H&E staining of the tumour tissues, livers and kidneys of mice from each group. (F‐I) Immunofluorescence analysis of Ki‐67, MELK, p‐ATM and p53 in each group of mouse tumour tissues, * *P* < .05; ** *P* < .01; *** *P* < .001

UMUC3 cells (1 × 10^6^ per mouse) were subcutaneously injected into the dorsal of BALB/c Nude mice. When the tumours just could be seen (approximately 2 weeks later), all mice were divided into three groups (NC group, low dose injection group/D1 group, high dose injection group/D2 group; 4 mice per group). The DMSO was injected into the abdominal cavity of NC group mice; OTSSP167 was injected into the abdominal cavity of injection group mice at concentrations of 2 µg/g and 6 µg/g, respectively. The injection operation was conducted 100 µL/mouse at a frequency of once every day (Figure 7A). The continuous measurement of tumour growth activity was conducted as previously mentioned. Compared with that of the NC group, the tumour growth of the OTSSP167 injection group mice was significantly inhibited. There was no significant difference in bodyweight of each group of mice; however, the dissected‐tumour weight in the OTSSP167 injection group was lighter (Figure 7B‐C). All of these significant differences were dose‐dependent. H&E staining indicated that the OTSSP167 injection group tumour tissues contained fewer tumour cells than did those of the NC group (Figure 7D). In addition, the livers and kidneys of mice in each group did not show drug‐induced damage (Figure 7E). It preliminarily confirmed the safety of OTSSP167 as a drug for inhibiting bladder cancer. Moreover, immunofluorescence analysis of mouse tumour tissues demonstrated that the number of cells with MELK and Ki‐67 positive staining was lower in the OTSSP167 injection group than in the control group, while the number of cells with p‐ATM and p53 positive staining was higher (Figure 7F‐I), which was consistent with the in vitro and in vivo MELK inhibition results.

### 3.8 OTSSP167 restored the up‐regulation effect of the MELK plasmid

**Figure 8:**
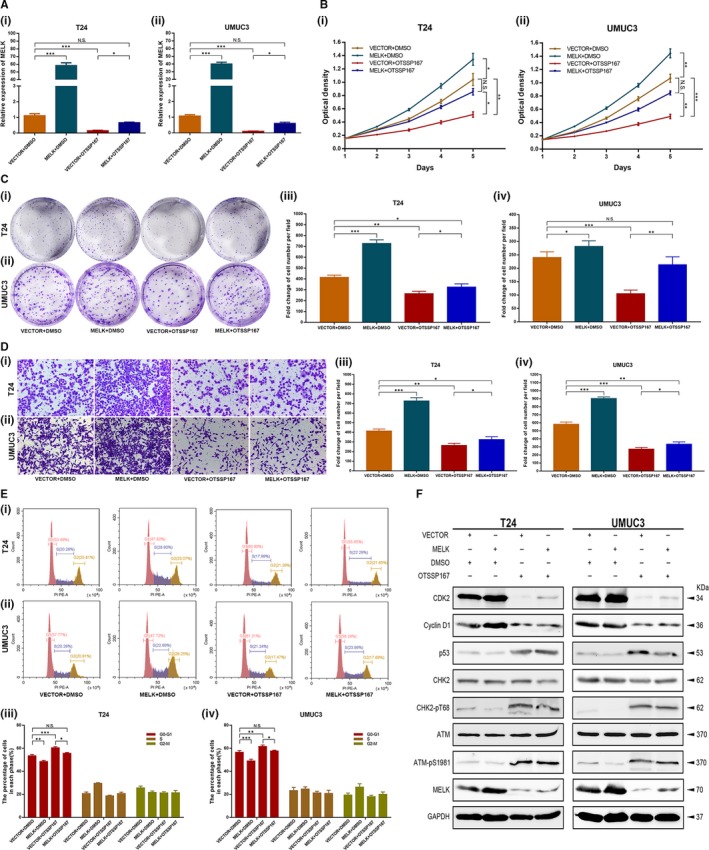
OTSSP167 restored the up‐regulation effect of the MELK plasmid. A, The expression of MELK was up‐regulated at transcription level with MELK plasmid; it was down‐regulated by OTSSP167. OTSSP167 could restore the up‐regulation effect caused by transfection with the MELK plasmid. (B‐D) Cellular functional studies, MTT (B), clonogenic forming (C) and migration (D) assays confirmed the restoration function of OTSSP167. E, OTSSP167 restored the cell cycle effect caused by transfection with the MELK plasmid. F, OTSSP167 restored the inhibition effect of the ATM/CHK2/p53 pathway, which was caused transfection with the MELK plasmid, * *P* < .05; ** *P* < .01; *** *P* < .001

To further study the restoration function of OTSSP167, T24 and UMUC3, cells were treated with one of four sets of conditions: VECTOR + DMSO, MELK + DMSO, VECTOR + OTSSP167 and MELK + OTSSP167. Consistent with the results mentioned above, the expression of MELK was up‐regulated at the mRNA level by transfection with the MELK plasmid; in contrast, it was down‐regulated by treatment with OTSSP167. Interestingly, OTSSP167 treatment restored the up‐regulation effect of the MELK plasmid (Figure 8A). Further cellular functional experiments, such as MTT (Figure 8B), clonogenic (Figure 8C) and migration (Figure 8D) assays, confirmed the restoration function of OTSSP167. Flow cytometry analysis showed that compared with the cells in the VECTOR + DMSO transfected group, the proportion of cells in the G1/S phase was significantly decreased in the MELK + DMSO transfected group and was markedly increased in the VECTOR + OTSSP167 transfected group; however, no obvious change was seen in the MELK + OTSSP167 group (Figure 8E). These results indicated that OTSSP167 restored the inhibitory effect of the ATM/CHK2/p53 pathway, which was caused by transfection with the MELK plasmid. Next, we detected the expression changes of several key proteins, such as MELK, ATM, CHK2, p53, CDK2 and cyclin D1. The Western blot results revealed the same kinds of changes that were seen in previous results (Figure 8F). This result proves our previous supposition.

## 4 DISCUSSION

Several studies have reported that MELK acts as an oncogene in many kinds of tumours from multiple systems.^33^, ^34^, ^35^, ^36^, ^37^, ^38^ In this study, we confirmed the oncogenic role of MELK in bladder tumorigenesis and elucidated for the first time the regulatory mechanism of MELK inhibition in BCa cells by bioinformatics analysis as well as in vitro/ in vivo investigation. Moreover, we have proved the anti‐tumour effects of OTSSP167 in bladder cancer.

The tumorigenesis function of MELK has been reported in many studies, but it has never been studied in BCa. First, the MELK expression level in bladder cancer was detected. Compared with the expression level in the normal bladder epithelial cell lines, the MELK mRNA expression level in the BCa cell lines was significantly up‐regulated. qRT‐PCR analysis also indicated that MELK mRNA was overexpressed in BCa tissues. More importantly, further analysis indicated that the expression levels of MELK mRNA were positively related to the tumour stage and tumour grade in BCa. Previous studies in other tumours have also confirmed the correlation of MELK and tumour stage and/or tumour grade.^26^, ^33^ As external validation, large sample data from the TCGA database confirmed the result of MELK expression in our tissue samples at the mRNA level. Further analyses of the genomic microarray profile http://www.ncbi.nlm.nih.gov/geo/query/acc.cgi?acc=GSE13507 showed that MELK was an independent prognostic factor of BCa and was significantly correlated with gender, age, T stage, N stage, tumour grade and progression, and high expression of MELK predicted poor prognosis (CSS and OS) in BCa patients. Consistently, the cohort study further proved that MELK expression in BCa tissues was up‐regulated at the mRNA level and was correlated with tumour progression. In addition, in accordance with bioinformatics analyses of http://www.ncbi.nlm.nih.gov/geo/query/acc.cgi?acc=GSE13507, MELK was involved in the cell cycle, G1/S transition of the mitotic cell cycle, DNA repair and replication, which was similar to previous reports.^6^, ^26^, ^33^, ^39^, ^40^, ^41^, ^42^, ^43^

Next, loss‐of‐function and gain‐of‐function assays were performed to elucidate the regulatory functions of MELK in BCa. MELK silencing demonstrated a marked inhibitory effect on BCa cells by reducing proliferation, inducing G1/S cell cycle arrest and attenuating cell migration. These tumour biological functions were markedly enhanced in cells when MELK was overexpressed in them. Interestingly, the positive proliferation and cell migration functions of MELK in tumours have been demonstrated in previous studies.^12^, ^21^, ^22^, ^24^, ^44^ Nevertheless, the role of MELK in the cell cycle remains controversial. MELK has been reported as an important regulator of the G2/M transition in many studies.^16^, ^22^, ^23^, ^24^ In contrast, MELK silencing was found to contribute to the delay of S phase progression by Beke et al and Kig et al.^13^, ^30^ Our study revealed that MELK may act as a negative regulator of the G1/S transition, which was consistent with Beke et al and Kig et al To investigate the reason that MELK plays a different role in cell cycle progression according to previous studies, cell cycle assays of other cancer cell lines, such as liver cancer and kidney cancer, were assessed following MELK being knocked down. MELK silencing demonstrated G1/S cell cycle arrest in p53 mutant cell lines (Huh7, 786O), which was similar to what was observed in the p53 mutant BCa cell lines T24 and UMUC3, but there was G2/M cell cycle arrest in p53 wild type cells (HepG2, 769P, Figure S3). Owing to the lack of additional tumour cell lines, we could not conclude that p53 affects the role of MELK in the cell cycle. However, it is an indisputable fact that p53 pathway defects contribute to diagnosis and therapy difficulties as well as adverse clinical outcomes in BCa. Moreover, MELK has been shown in some studies to promote tumorigenesis via the p53 pathway.^13^, ^23^, ^25^, ^30^, ^31^, ^32^

Previous investigations suggested that MELK inhibition could lead to the successive phosphorylation of ATM and CHK2 and the up‐regulation of p53 and p21.^30^, ^31^, ^32^ This may be the reason that MELK silencing caused G1/S cell cycle arrest. We further detected changes in these cell cycle‐related genes in addition to ATM/CHK2/p53 pathway‐related genes at the transcriptional level and translation level when MELK was down‐regulated. The qRT‐PCR analysis demonstrated that MELK could regulate TP53, CDKN1A, CCND1 and CDK2, but it has not been observed that MELK significantly influenced ATM and CHK2 at the transcriptional level. Consistent with previous research, Western blot analysis revealed that down‐regulated MELK might activate phosphorylation of ATM and CHK2 to regulate the expression of p53 and p21, while down‐regulated MELK reduced MDMX, cyclin D1, CDK2 and E2F1 expression. When MELK was up‐regulated, phosphorylation of ATM and CHK2 decreased. Up‐regulated MELK activated the expression of MDMX, cyclin D1, CDK2 and E2F1, while it inhibited p53 and p21 expression. The linear relationship between MELK and these genes in the GEPIA database confirmed our Western blot results. Overall, MELK promoted BCa cell vitality by accelerating the transition out of G1/S phase via inhibiting the ATM/CHK2/p53 pathway in BCa cells.

**Figure 9:**
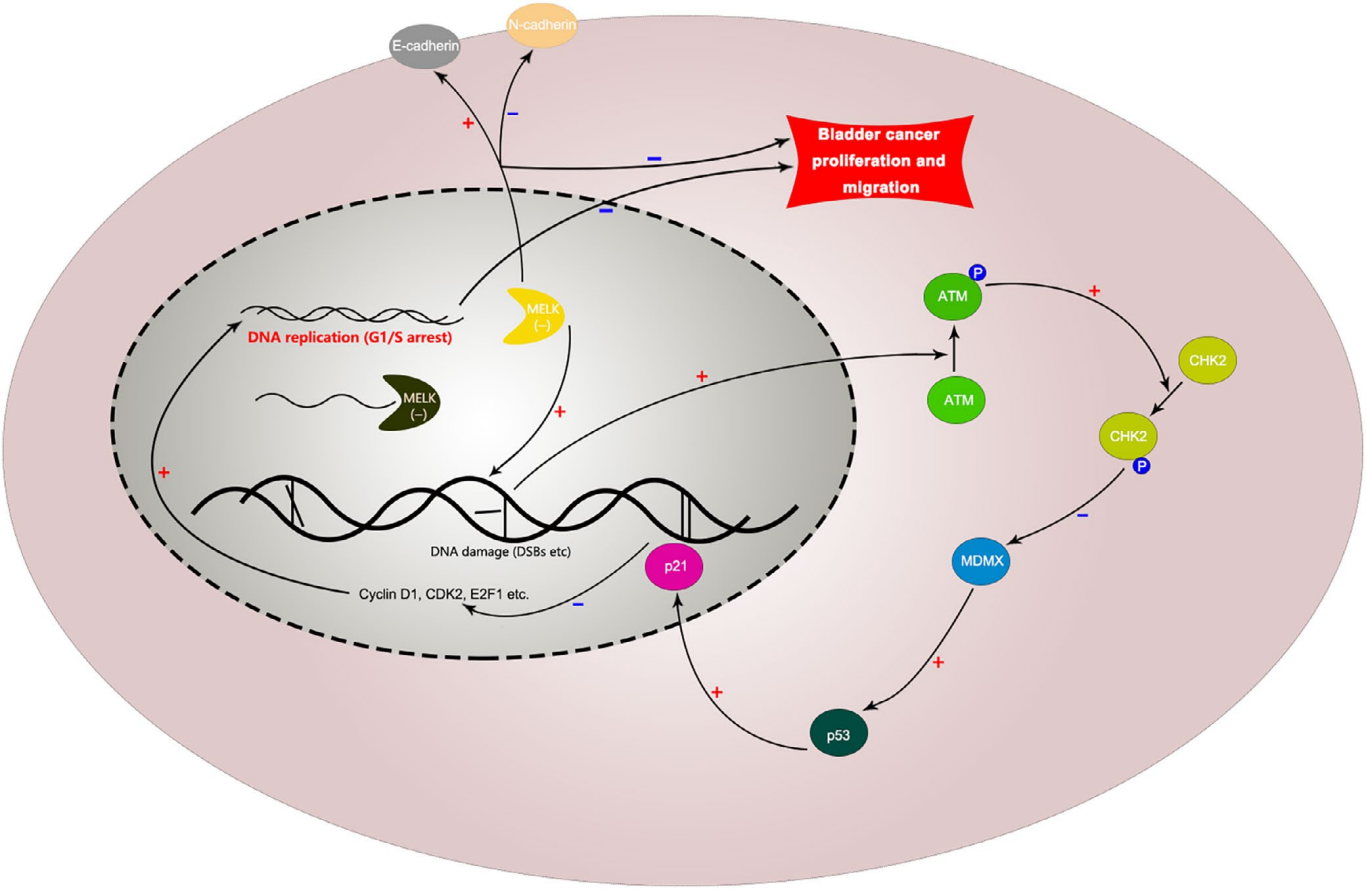
Mechanism diagram. Inhibition of MELK could reduce BCa cell proliferation and migration. Specifically, the down‐regulation of MELK could restrain the repair of DSBs and then activate the ATM/CHK2/p53 pathway, further inducing G1/S cell cycle arrest and leading to the suppression of BCa cell proliferation. In addition, the down‐regulation of MELK could suppress the EMT pathway to reduce the migration of BCa cells

ATM is an important gene involved in the DNA damage response. The ATM protein can detect DNA breaks and is involved in the activation, regulation of various cell cycle regulators and repair of DNA damage.^45^, ^46^, ^47^ CHK2 is a tumour suppressor gene encoding a serine/threonine kinase. It plays a very important role in the regulation of cell cycle checkpoints caused by DNA damage and participates in maintaining genome stability.^48^, ^49^, ^50^ MDMX, as an inhibitor of p53, could cause the ubiquitination and degradation of p53.^51^, ^52^, ^53^ CHK2 could inhibit the activity of MDMX and then rescue the inhibition of p53.^53^, ^54^ The ATM‐CHK2 pathway is activated when DSBs occur. MELK was proven to be associated with mitotic progression and DNA damage.^8^, ^9^, ^10^, ^11^, ^12^, ^13^ Kig et al confirmed that MELK was required for the repair of DNA damage (including double‐strand breaks, DSBs) in unperturbed replication.^30^ Squatrito et al reported that the loss of ATM/Chk2/p53 pathway components accelerated tumour development and contributed to radiation resistance in gliomas.^32^ In this study, the down‐regulation of MELK restrained the repair of DSBs and led to the continuous phosphorylation of ATM and CHK2. Then, the activated ATM/CHK2/p53 pathway, which up‐regulated p53 and p21, further induced G1/S cell cycle arrest and led to the suppression of BCa cell proliferation (Figure 9).

Furthermore, we investigated the effect of MELK down‐regulation on BCa cell growth in vivo. The continuous measurement of tumour growth activity and the weighing of dissected tumours revealed that MELK inhibition significantly suppressed BCa growth in vivo compared with what was observed in control group mice. Importantly, Western blot analyses were conducted to detect total protein and phosphorylated protein in mouse tumours. Immunofluorescence analysis of mouse tumours demonstrated that the number of MELK‐, Ki‐67‐ and CDK2‐positive cells was lower in the MELK inhibition group than it was in the control group, while the number of cells with p‐ATM‐, p53‐ and p21‐positive staining was higher. These results further confirmed the ATM/CHK2/p53 pathway results from the in vitro experiments.

Consistent with the experimental results of MELK silencing by siRNA or shRNA, OTSSP167, an inhibitor targeting MELK, displayed anti‐tumour activity against BCa cells both in vitro and in vivo. We observed that OTSSP167 exerted therapeutic potential via thwarting the proliferation and cell cycle progression of BCa cell lines. In addition, OTSSP167 significantly inhibited the tumour growth activity of mice in a dose‐dependent manner. Moreover, the livers and kidneys of mice in each group did not show drug‐induced damage, which preliminarily confirmed the safety of OTSSP167 as a drug for inhibiting bladder cancer. In addition, immunofluorescence analysis showed that treatment with OTSSP167 could up‐regulate the ATM/p53 signalling pathway, which was similar to what was observed following the inhibition of MELK. Functional studies in cells confirmed the corrective function of OTSSP167. Flow cytometry analysis indicated that OTSSP167 restored the cell cycle effect caused by the MELK plasmid. Western blot results of several key proteins, such as MELK, ATM, CHK2, p53, CDK2 and cyclin D1, revealed the same trend that was observed in previous results. This result proves our previous supposition. Therefore, ATM, CHK2 and p53 were confirmed to be crucial regulators related to the potency of MELK, acting as a negative regulator of the G1/S transition.

In summary, MELK overexpression predicts a poor prognosis in BCa patients. Both in vitro and in vivo studies indicated that MELK silencing with siRNA/shRNA or OTSSP167 treatment exhibited anti‐tumour effects through abrogating cell proliferation and migration. Furthermore, MELK can induce G1/S cell cycle arrest in BCa via activating the ATM/CHK2/p53 pathway.

## CONFLICTS OF INTEREST

The authors declare no conflict of interests.

## AUTHORS’ CONTRIBUTIONS

SC, QZ, ZG, YX and XW conceived and designed the study; SC, QZ, ZG, YW and LW performed the analysis procedures; SC, QZ, ZG, ML and LJ analysed the results; SC, QZ, YW and ML contributed analysis tools; SC, QZ, LW and LJ contributed to the writing of the manuscript. All authors reviewed the manuscript.

## Data Availability

The data sets used and/or analysed during the current study are available from the corresponding authors on reasonable request.
