# Corrigendum

**DOI:** 10.1111/jcmm.17423

**Published:** 2022-07-06

In Song Chen et al,^1^ there was an error in the figure panel labelling in Figure 7B(ii) and an image assembly error in Figure 8D(ii). The correct legends and figures are shown below. The authors confirm that all results and conclusions of this article remain unchanged.

**FIGURE 7:**
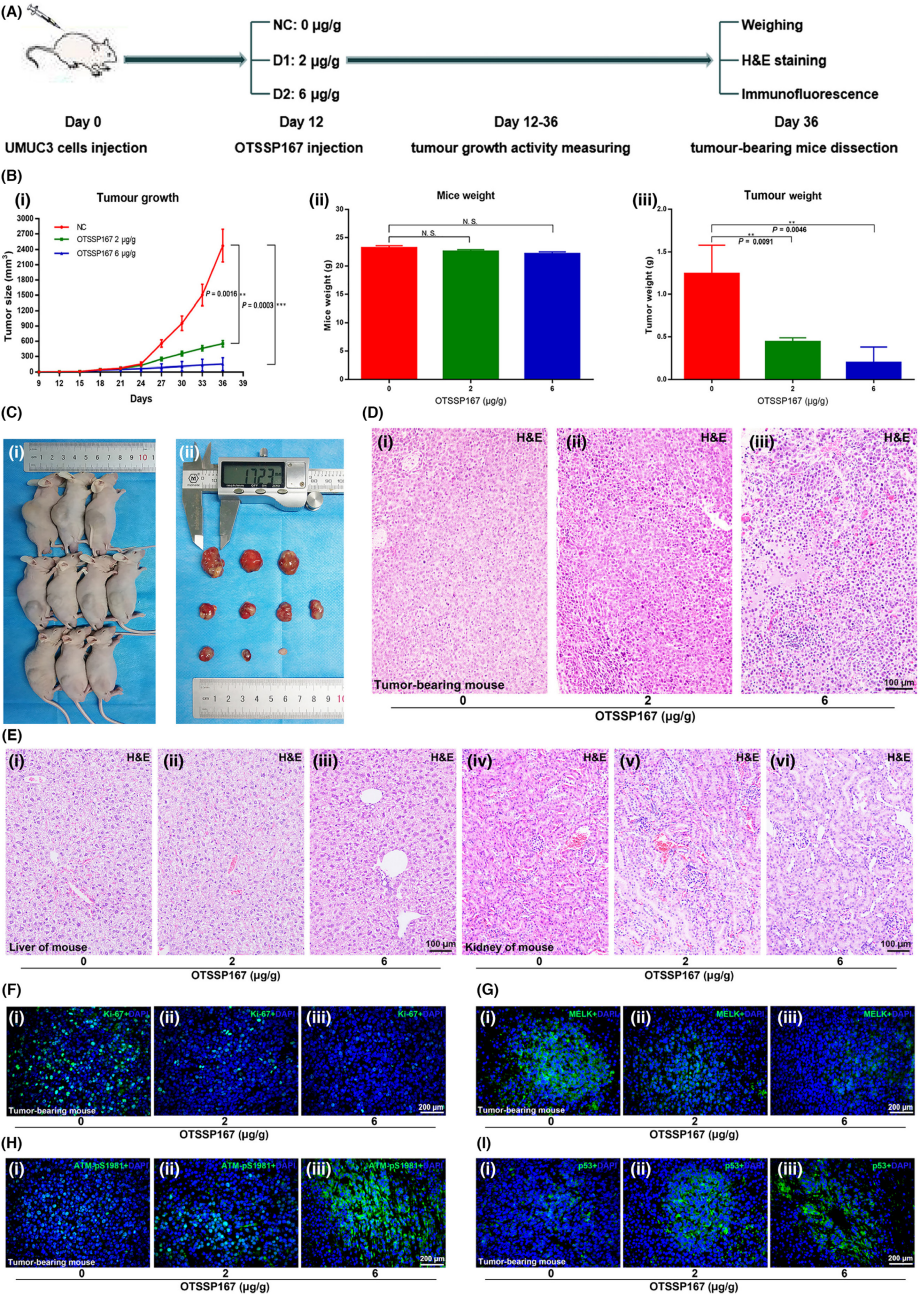
Inhibition of MELK by OTSSP167 results in potent anti‐tumour effects in bladder cancer in vivo. (A) The OTSSP167 injection anti‐tumour experiment performed with a mouse xenograft model is shown. (B, C) The measurement of tumour growth activity and mouse bodyweight as well as analysis of the dissected tumours. (D, E) Haematoxylin and eosin staining of the tumour tissues, livers and kidneys of mice from each group. (F–I) Immunofluorescence analysis of Ki‐67, MELK, p‐ATM and p53 in each group of mouse tumour tissues, *p < 0.05; **p < 0.01; ***p < 0.001

**FIGURE 8:**
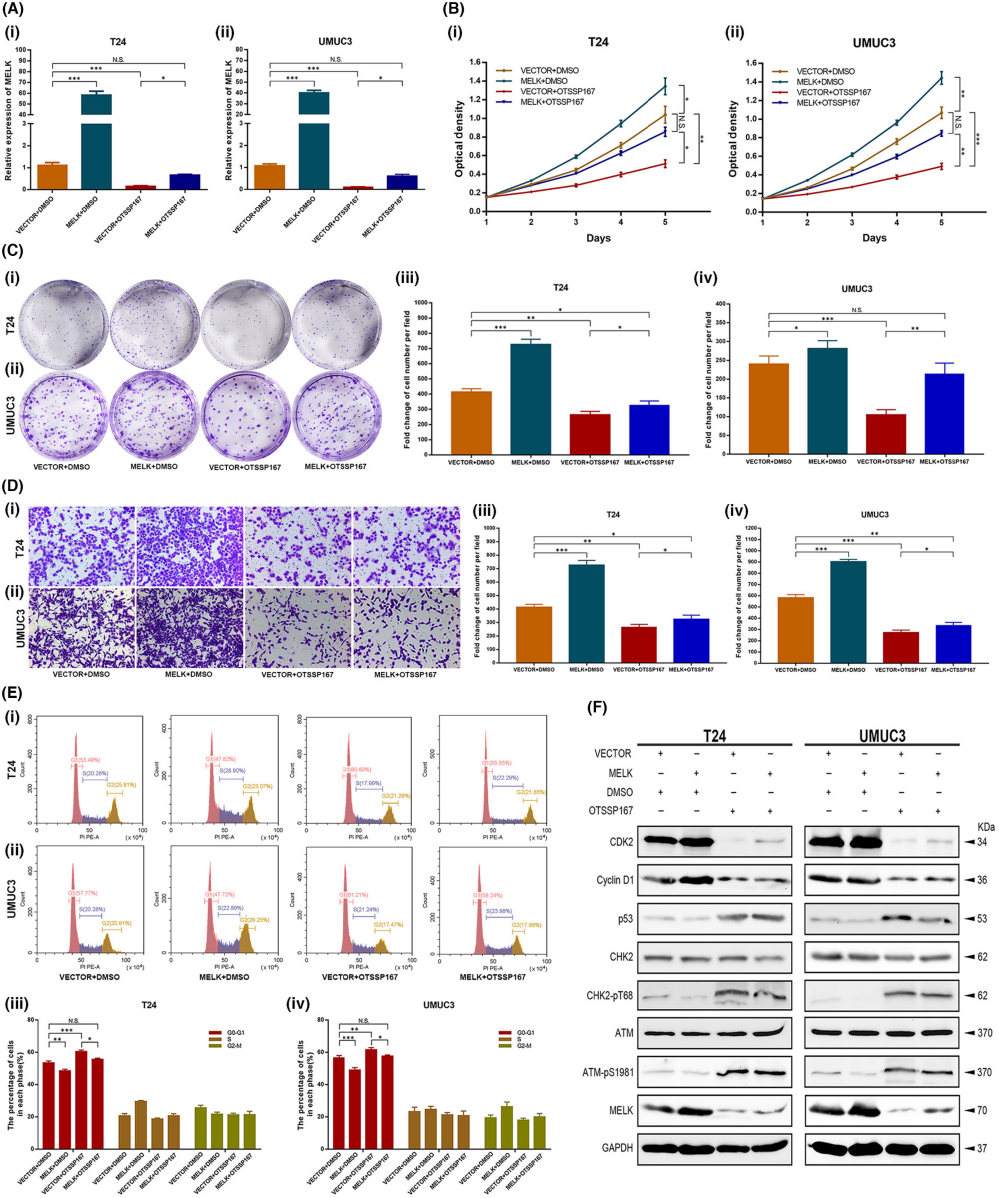
OTSSP167 restored the up‐regulation effect of the MELK plasmid. (A) The expression of MELK was up‐regulated at transcription level with MELK plasmid; it was down‐regulated by OTSSP167. OTSSP167 could restore the up‐regulation effect caused by transfection with the MELK plasmid. (B–D) Cellular functional studies, MTT (B), clonogenic forming (C) and migration (D) assays confirmed the restoration function of OTSSP167. E, OTSSP167 restored the cell cycle effect caused by transfection with the MELK plasmid. (F) OTSSP167 restored the inhibition effect of the ATM/CHK2/p53 pathway, which was caused transfection with the MELK plasmid, *p < 0.05; **p < 0.01; ***p < 0.001
