# Supplementary material for: Inhibition of MELK produces potential anti‐tumour effects in bladder cancer by inducing G1/S cell cycle arrest via the ATM/CHK2/p53 pathway
Source: J Cell Mol Med. 2019 Dec 10;24(2):1804–21. doi: 10.1111/jcmm.14878 (PMC6991658; doi:10.1111/jcmm.14878)
Supplement: Supplementary file 1 [file JCMM-24-1804-s001.pdf]

Supplementary Information 1

Supplementary Figures

A

Cox analyses of overall survival among 165 bladder cancer patients

Bladder cancer patients (n = 165)	Univariate analysis			Multivariate analysis		
	HR	95% CI	p value	HR	95% CI	p value
**MELK expression** (low/high)	1.762	1.092-2.842	0.020	1.384	1.021-1.895	0.038
**Gender** (female/male)	0.631	0.356-1.120	0.116	-	-	-
**Age**	1.069	1.044-1.095	<0.001	1.086	1.056-1.117	<0.001
**T Stage**	2.017	1.609-2.527	<0.001	1.057	0.762-2.158	0.176
**N Stage** (N1-N3 vs. N0)	7.701	4.011-14.788	<0.001	2.440	1.077-5.528	0.033
**M Stage** (M1 vs. M0)	9.898	4.378-22.378	<0.001	5.200	1.803-14.997	0.002
**Grade** (low/high)	2.695	1.670-4.347	<0.001	0.892	0.488-1.629	0.710
**Recurrence** (no/yes)	1.417	0.725-2.771	0.308	-	-	-
**Progression** (no/yes)	2.927	1.779-4.815	<0.001	3.250	1.846-5.723	<0.001

B

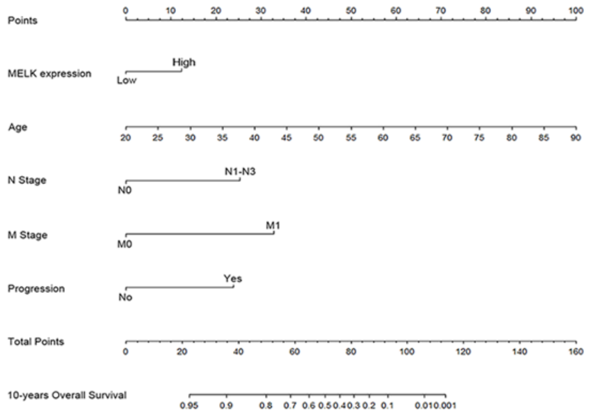

C

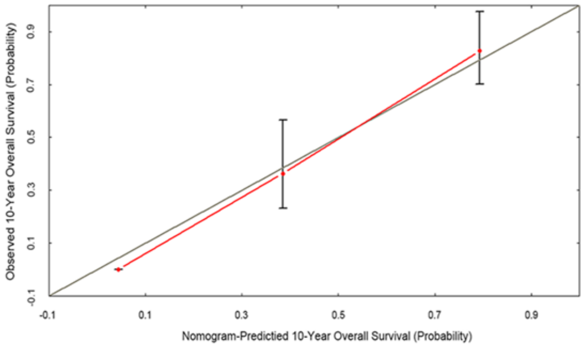

Supplementary Figure S1. MELK was associated with poor overall survival. (A) Cox analyses of overall survival among 165 bladder cancer patients. **(B)** The nomogram for 10-year overall survival prediction of BCa patients. **(C)** The calibration curves developed for 10-year overall survival prediction nomogram.

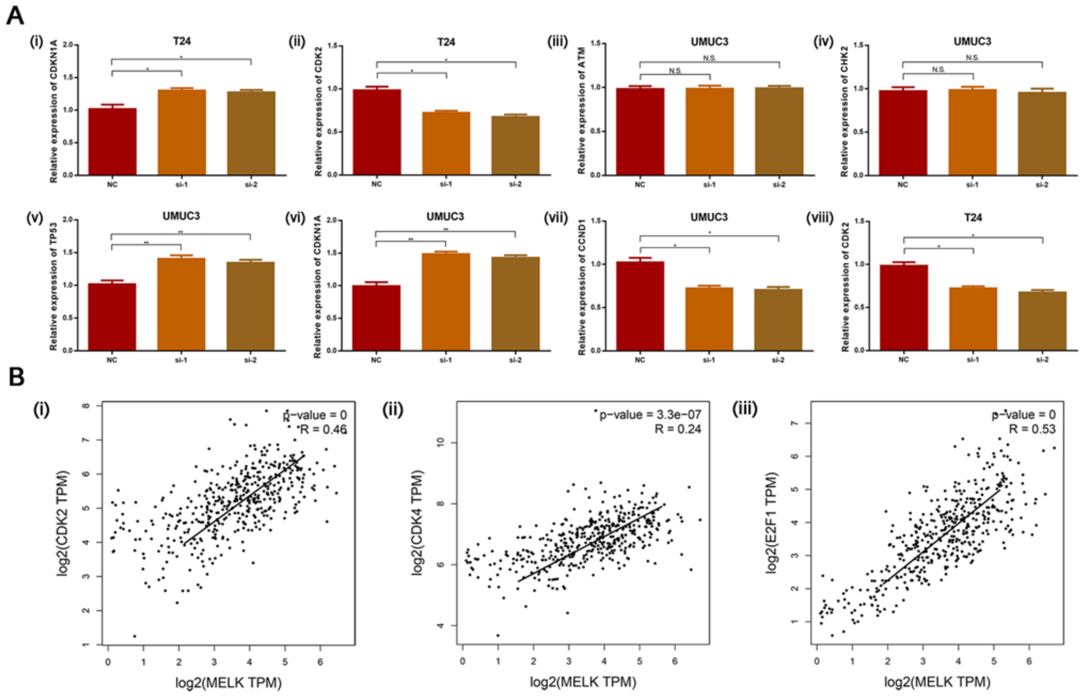

Supplementary Figure S2. The correlation between MELK and G1/S phase cell cycle-related genes as well as ATM/CHK2/p53 pathway-related genes. (A) The correlation between MELK and G1/S phase cell cycle-related genes as well as ATM/CHK2/p53 pathway-related genes in transfected BCa cells was investigated with qRT-PCR analysis. **(B)** The linear relationship between MELK and G1/S phase cell cycle-related genes in GEPIA database, * $p < 0.05$; ** $p < 0.01$.

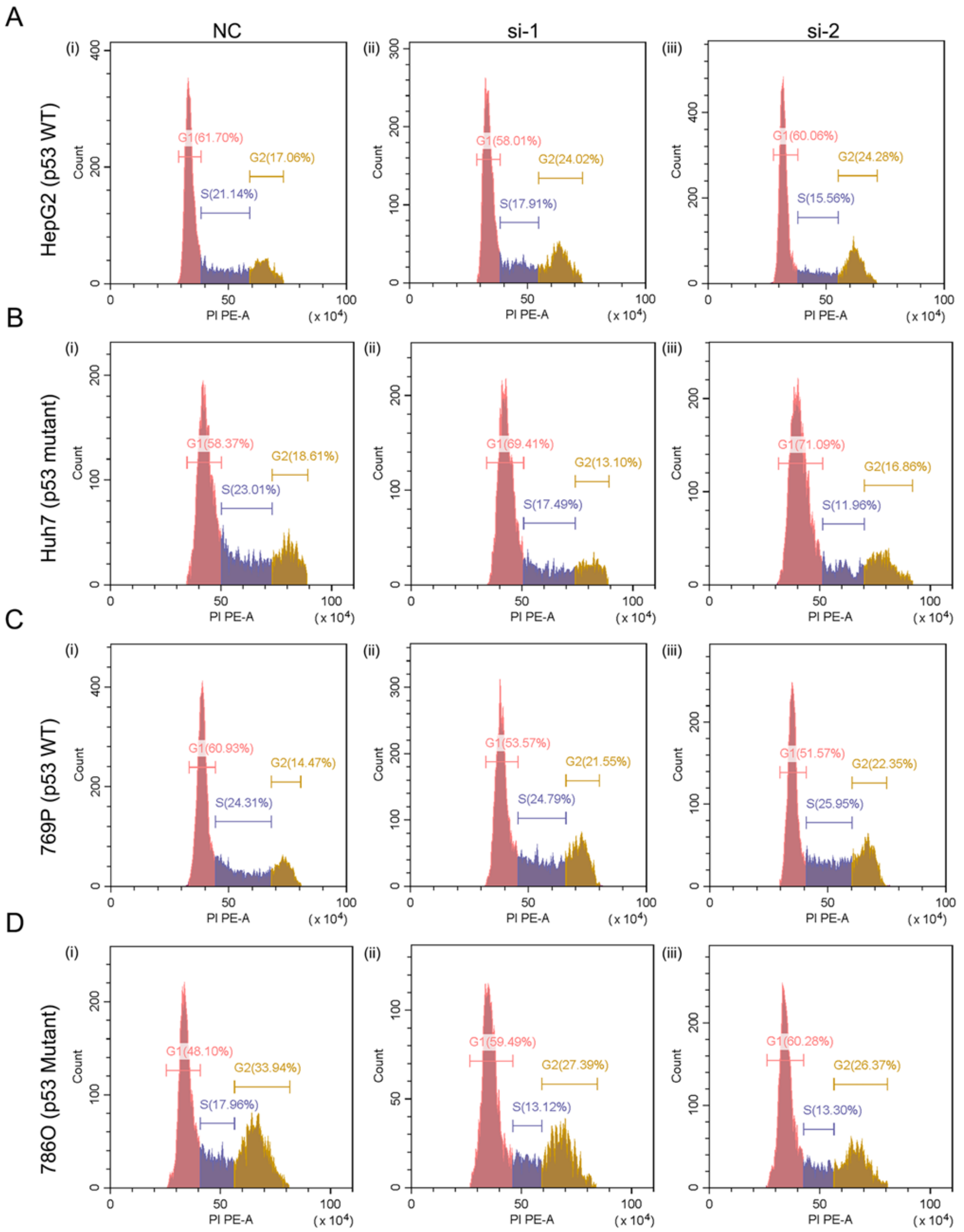

Supplementary Figure S3. MELK silencing induced different cell cycle arrest in different cancer cells. (A) MELK silencing in HepG2 cells (p53 wild type liver cancer cell line) induced cell cycle arrest at G2/M phase. **(B)** MELK silencing in Huh7 cells (p53 mutant liver cancer cell line) induced cell cycle arrest at G1/S phase. **(C)** MELK silencing in 769P cells (p53 wild type kidney cancer cell line) induced cell cycle arrest at G2/M phase. **(D)** MELK silencing in 786O cells (p53 mutant kidney cancer cell line) induced cell cycle arrest at G1/S phase.

Supplementary Information 2

Supplementary Tables

Supplementary Table S1. List of primers for qRT-PCR.

Gene name	Symbol	Forward primer	Reverse primer	Annealing Temperature (°C)	Length (bp)
Ataxia telangiectasia mutated	*ATM*	ATCTGCTGCCGTCAACTAGAA	GATCTCGAATCAGGCGCTTAAA	57	82
Cyclin D1	*CCND1*	GCTGCGAAGTGGAAACCATC	CCTCCTTCTGCACACATTTGAA	56	135
Cyclin dependent kinase 2	*CDK2*	CCAGGAGTTACTTCTATGCCTGA	TTCATCCAGGGGAGGTACAAC	56	90
Cyclin dependent kinase inhibitor 1A	*CDKN1A*	TGTCCGTCAGAACCCATGC	AAAGTCGAAGTTCCATCGCTC	58	139
Checkpoint kinase 2	*CHK2*	TGAGAACCTTATGTGGAACCCC	ACAGCACGGTTATACCCAGC	58	82
Glyceraldehyde-3-phosphate dehydrogenase	*GAPDH*	GAAGGTGAAGGTCGGAGTC	GAAGATGGTGATGGGATTTC	56	197
Maternal embryonic leucine zipper kinase	*MELK*	TCTCCCAGTAGCATTCTGCTT	TGATCCAGGGATGGTTCAATAGA	56	94
Tumor protein p53	*TP53*	CAGCACATGACGGAGGTTGT	TCATCCAAATACTCCACACGC	58	125

Supplementary Table S2. List of primary antibodies.

Antigens	Species antibodies raised in	Dilution (IF)	Dilution (WB)	Dilution (IHC)	Supplier
CDK2, human	Rabbit, monoclonal	1:200	1:2,000	-	Abcam, UK, Cat. #ab32147
Cyclin D1, human	Rabbit, monoclonal	-	1:2,000	-	Cell Signaling Technology, USA, Cat. #2978
GAPDH, human	Mouse, monoclonal	-	1:2,000	-	Santa Cruz Biotechnology Inc., USA, Cat. #sc-365062
Ki-67, human	Rabbit, monoclonal	1:200	2ug/ml	-	Novus Biologicals, USA, Cat. #NBP2-19012
MDMX, human	Rabbit, monoclonal	-	1:500	-	Proteintech, China, Cat. #17914-1
MELK, human	Rabbit, monoclonal	1:100	1:500	1:100	Proteintech, China, Cat. #11403-1
p21, human	Rabbit, monoclonal	1:100	1:1,000	-	Abcam, UK, Cat. #ab109520
p53, human	Rabbit, monoclonal	1:200	1:1,000	-	Abcam, UK, Cat. #ab183544
E2F1, human	Rabbit, monoclonal	-	1:2,000	-	Abcam, UK, Cat. #ab179445
E-Cadherin, human	Rabbit, monoclonal	-	1:2,000	-	Abcam, UK, Cat. #ab76055
N-Cadherin, human	Rabbit, monoclonal	-	1:2,000	-	Abcam, UK, Cat. #ab76011
ATM, human	Rabbit, monoclonal	-	1:1,000	-	Abcam, UK, Cat. #ab32420
Chk2, human	Rabbit, monoclonal	-	1:5,000	-	Abcam, UK, Cat. #ab109413
ATM (phospho S1981), human	Rabbit, monoclonal	1:500	1:2,0000	-	Abcam, UK, Cat. #ab81292
Chk2 (phospho T68), human	Rabbit, monoclonal	-	1:2,000	-	Abcam, UK, Cat. #ab32148

Supplementary Table S3. List of secondary antibodies and counterstaining of nuclei.

Secondary detection system used	Host	Method	Dilution	Supplier
Anti-Mouse-IgG (H+L)-HRP	Goat	WB	1:10,000	Sungene Biotech, China, Cat. #LK2003
Anti-Rabbit-IgG (H+L)-HRP	Goat	WB	1:10,000	Sungene Biotech, China, Cat. #LK2001
Anti-rabbit IgG (H+L), F(ab')2 Fragment (Alexa Fluor® 488 Conjugate)	Goat	WB	1:50	Cell Signaling Technology, USA, Cat. #4412
Anti-mouse IgG (H+L), F(ab')2 Fragment (Alexa Fluor® 555 Conjugate)	Goat	WB	1:50	Cell Signaling Technology, USA, Cat. #4408
Anti-goat IgG-FITC	Rabbit	IF	1:100	Boster Biological Technology, China, Cat. #BA1110
Anti-goat IgG-Cy3	Rabbit	IF	1:100	Boster Biological Technology, China, Cat. #BA1034
Hoechst 33342 nucleic acid staining (DAPI)	-	IF	1:750	Molecular Probes/Invitrogen, USA, Cat. #A11007

Supplementary Table S4. The clinicopathological information of 31 bladder cancer patients.

Patient number	Gender	Age	Tumor stage	Tumor Grade	Lymphnodes status	Infiltration
1	Male	63	T2	G1	-	MIBC
2	Male	48	T3	G3	-	MIBC
3	Male	74	T4	G3	+	MIBC
4	Female	59	T3	G2	-	MIBC
5	Male	62	T3	G3	+	MIBC
6	Male	72	T1	G3	-	NMIBC
7	Male	64	T3	G3	+	MIBC
8	Male	66	T1	G1	-	NMIBC
9	Male	52	T4	G2	+	MIBC
10	Male	54	T2	G1	-	MIBC
11	Male	52	T1	G3	-	NMIBC
12	Male	73	T3	G2	-	MIBC
13	Female	63	T1	G3	-	NMIBC
14	Male	61	T2	G1	-	MIBC
15	Female	68	T3	G3	+	MIBC
16	Male	56	T2	G1	-	MIBC
17	Male	48	T2	G2	-	MIBC
18	Male	61	T3	G2	-	MIBC
19	Male	64	T1	G1	-	NMIBC
20	Male	70	T2	G2	-	MIBC
21	Male	26	T2	G1	-	MIBC
22	Male	51	T2	G2	-	MIBC
23	Male	56	T3	G1	-	MIBC
24	Female	69	T2	G1	-	MIBC
25	Male	67	T1	G3	-	NMIBC
26	Male	76	T2	G1	-	MIBC
27	Male	79	T3	G3	+	MIBC
28	Male	62	T2	G1	-	MIBC
29	Male	58	T1	G2	-	NMIBC
30	Male	66	T2	G1	-	MIBC
31	Female	71	T2	G3	-	MIBC

Supplementary Table S5. Pathways associated with MELK expression in bladder cancer.

Gene set	ES	NES	NOM p-val	FDR q-val
Cell cycle	0.7386	1.6625	0.0000	0.0981
Oocyte meiosis	0.6408	1.6858	0.0000	0.2064
DNA replication	0.8222	1.5854	0.0000	0.1212
Proteasome	0.7833	1.6766	0.0000	0.1226
Progesterone mediated oocyte maturation	0.5771	1.6556	0.0000	0.0793
N-glycan biosynthesis	0.6701	1.5876	0.0078	0.1371
Spliceosome	0.6339	1.5802	0.0098	0.1128
Homologous recombination	0.7459	1.6250	0.0117	0.1006
Mismatch repair	0.7549	1.5053	0.0165	0.2019
Cysteine and methionine metabolism	0.5743	1.5321	0.0252	0.1920
Selenoamino acid metabolism	0.5880	1.4678	0.0257	0.2011
Vibrio cholerae infection	0.6048	1.5197	0.0304	0.2040
Terpenoid backbone biosynthesis	0.7344	1.5007	0.0313	0.1963
Steroid biosynthesis	0.7537	1.5178	0.0318	0.1894
Nucleotide excision repair	0.6155	1.4900	0.0359	0.2060
Biosynthesis of unsaturated fatty acids	0.7336	1.4769	0.0373	0.2075
RNA degradation	0.5759	1.4699	0.0405	0.2092
Protein export	0.7036	1.4809	0.0408	0.2126
Pyrimidine metabolism	0.5478	1.4570	0.0426	0.2017
Prostate cancer	0.4920	1.4105	0.0460	0.2519
Pentose phosphate pathway	0.6858	1.4676	0.0472	0.1908

Supplementary Information 3

Supplementary material and methods

Cell proliferation, clonogenic forming and migration assay

We performed MTT assay to determine cell proliferation. The transfected BCa cells were plated into 96-well plates in 200 μl medium to culture for 5 days, and then were added into 20 μl MTT solution (5 mg/ml) each well, incubated for 4 h. Microplate reader (Cat. #SpectraMax M2, Molecular Devices, USA) was measured the absorbance at 490m.

In the clonogenic forming assay, the transfected BCa cells were plated in 6-well plates to culture for 2 weeks (1000 cells per well). The cells were fixed with 4% paraformaldehyde for 30 min after washing gently twice by PBS, and then stained with crystal violet. The colonies number was calculated.

The transfected BCa cells were plated in 200 μl serumfree medium in the upper transwell chamber (Corning, USA). 600 μl medium containing 10% FBS was filled into the lower chamber. After culturing for 24 h, we removed the cells on the upper chamber. The cells on the lower chamber were fixed with 4% paraformaldehyde and stained with crystal violet. The number of stained cells was calculated under an inverted phase contrast microscope and photographed in 3 random fields.

Immunofluorescence staining, Hematoxylin and Eosin (H&E) Staining, Immunohistochemistry (IHC) staining

We seeded the transfected BCa cells on 12mm coverslips, and fixed the cells with 4%

paraformaldehyde. The Biofavor Biotech Ltd. (Wuhan, China) accomplished the immunofluorescence staining with the fixed BCa cells or tissue samples. We analyzed the immunofluorescence staining with a confocal microscope system (Nikon C2+ Confocal Microscope, Japan).

The mice tumor tissues were stained with Hematoxylin & Eosin. We deparaffinized and continuously rehydrated the sections by xylene, 100 % ethanol, 96 % ethanol, 80 % ethanol, 70 % ethanol and H_2O, and then stained the sections with 10 % Hematoxilin (Sigma-Aldrich). The cell nuclei were appeared after washing.1 % Eosin (Sigma-Aldrich) containing 0.2 % glacial acetic acid was used to stain the cytoplasm. The slides were quickly washed and dehydrated in 70 %, 80 %, 96 %, 100 % ethanol and xylene. We used the inverted phase contrast microscope (Cat. #DMI 1, Leica, Wetzlar, Germany) to photograph the sections.

For IHC staining, a proportion of surgical tissue specimens were fixed with formalin to for paraffin-embedded. IHC analyses were performed on 4 μm thick sections. Briefly, each slide was incubated with primary antibody against MELK overnight after a series of procedures (de-paraffin, antigen retrieval, rinse). This was followed by incubation with the anti-rabbit IgG-HRP antibody for 30 min. The membrane was then washed five times with TBST and enriched with the brown color of DAB Enhancer (Dako, China). The MELK expression was evaluated by three experienced pathologists. We used phase contrast microscope to analyze IHC sections.

Supplementary Information 4

Related File 1. Ethics Committee Approval (number: 2015029).

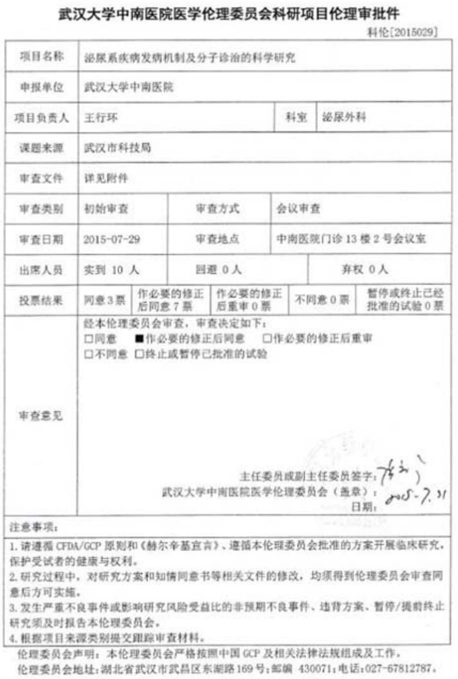

武汉大学中南医院医学伦理委员会科研项目伦理审批件

科伦[2015029]

项目名称	泌尿系疾病发病机制及分子诊治的科学研究				
申报单位	武汉大学中南医院				
项目负责人	王行环			科室	泌尿外科
课题来源	武汉市科技局				
审查文件	详见附件				
审查类别	初始审查	审查方式	会议审查		
审查日期	2015-07-29	审查地点	中南医院门诊 13 楼 2 号会议室		
出席人员	实到 10 人	回避 0 人		弃权 0 人	
投票结果	同意 3 票	作必要的修正后同意 7 票	作必要的修正后重审 0 票	不同意 0 票	暂停或终止已经批准的试验 0 票
审查意见	经本伦理委员会审查，审查决定如下：<br>☐同意 ■作必要的修正后同意 ☐作必要的修正后重审<br>☐不同意 ☐终止或暂停已批准的试验<br>主任委员或副主任委员签字：<br>武汉大学中南医院医学伦理委员会（盖章）：<br>日期：2015-7.31				

注意事项：

1. 请遵循 CFDA/GCP 原则和《赫尔辛基宣言》、遵循本伦理委员会批准的方案开展临床研究，保护受试者的健康与权利。
2. 研究过程中，对研究方案和知情同意书等相关文件的修改，均须得到伦理委员会审查同意后方可实施。
3. 发生严重不良事件或影响研究风险受益比的非预期不良事件、违背方案、暂停/提前终止研究须及时报告本伦理委员会。
4. 根据项目来源类别提交跟踪审查材料。

伦理委员会声明：本伦理委员会严格按照中国 GCP 及相关法律法规组成及工作。

伦理委员会地址：湖北省武汉市武昌区东湖路 169 号；邮编 430071；电话：027-67812787。

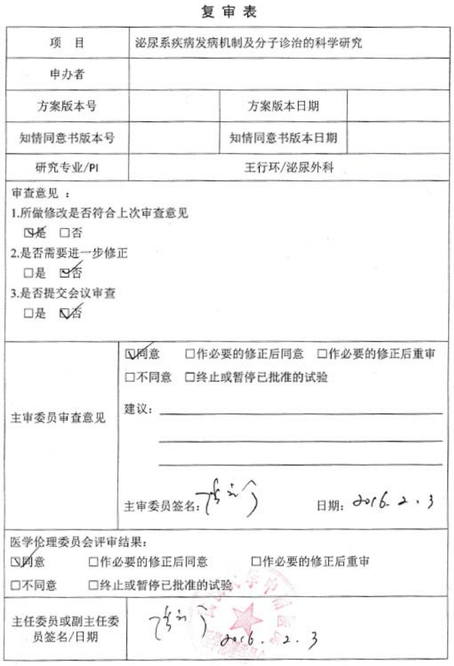

复 审 表

项 目	泌尿系疾病发病机制及分子诊治的科学研究		
申办者			
方案版本号		方案版本日期	
知情同意书版本号		知情同意书版本日期	
研究专业/PI	王行环/泌尿外科		

审查意见：

1.所做修改是否符合上次审查意见

☑是 ☐否

2.是否需要进一步修正

☐是 ☑否

3.是否提交会议审查

☐是 ☑否

主审委员审查意见：

☑同意 ☐作必要的修正后同意 ☐作必要的修正后重审

☐不同意 ☐终止或暂停已批准的试验

建议：

主审委员签名： 日期：2016.2.3

医学伦理委员会评审结果：

☑同意 ☐作必要的修正后同意 ☐作必要的修正后重审

☐不同意 ☐终止或暂停已批准的试验

主任委员或副主任委员签名/日期：2016.2.3

Approval by the Ethics Committee at Zhongnan Hospital of Wuhan University for the microarray and RT-PCR analysis using RNA isolated from human bladder cancer tissues and normal bladder tissues from donors by accidental death.
